# A Practitioner-Informed Decision Tree for Selecting Harmful Cyanobacteria Bloom Control and Mitigation Techniques

**DOI:** 10.1002/wat2.70005

**Published:** 2025-02-23

**Authors:** Desirée Tullos, Megan M. Skinner, Hans W. Paerl, Ellen P. Preece

**Affiliations:** 1 Department of Biological and Ecological Engineering, Oregon State University, Corvallis, Oregon, USA; 2 U.S. Fish and Wildlife Service, Klamath Falls Fish and Wildlife Office, Klamath Falls, Oregon, USA; 3 Department of Earth, Marine, and Environmental Sciences, University of North Carolina at Chapel Hill, Institute of Marine Sciences, Morehead City, North Carolina, USA; 4 California Department of Water Resources, West Sacramento, California, USA

**Keywords:** cyanobacteria, decision tree, harmful algal bloom mitigation and control, in-lake restoration methods, nutrient management

## Abstract

Harmful Cyanobacterial Blooms (HCBs) threaten ecological and human health, and their incidence and magnitude appear to be rising globally. However, a lack of guidance exists on how to choose the best HCB control and mitigation strategy for different types of water bodies. The portfolio of available in situ control techniques is diverse, ranging from experimental to well established, with complicated and poorly-documented records of effectiveness across different settings and a range of unintended ecological consequences. We introduce a decision tree that synthesizes current science and practitioner experience in a framework that can be used to examine conditions under which HCB control techniques are likely to be appropriate and most effective. The factors that establish branching and the classification of techniques within the decision tree were based on the review of peer-reviewed and gray literature, and on responses to a national survey. Key factors influencing the feasibility and effectiveness of HCB control include whether nutrient loads are sourced externally or internally, the size of the treatment area, and the vulnerability of and regulations governing the receiving water body. Survey results point to important regional differences in the application of HCB control techniques, whereas demonstration of the decision tree with real-world case studies highlights some of the practical issues managers face in making decisions about treatment techniques. Supporting Information provides a comprehensive review of current science, appropriate use, and costs for individual techniques.

## 1 | Introduction

Harmful Cyanobacterial Blooms (HCBs) threaten ecological and human health, and their incidence and magnitude appear to be rising globally (Fang et al. 2022; Ho and Michalak 2019; but see Wilkinson et al. 2022). The proliferation of HCBs has resulted in adverse ecological (Wurtsbaugh, Paerl, and Dodds 2019), economic (Dodds et al. 2009), and human health impacts (Codd et al. 2020). As HCBs continue to degrade more water bodies, there is a growing need to implement appropriate measures to control and mitigate blooms. The primary and most important solution to addressing HCBs is to decrease nutrient loading into waterways (Mucci et al. 2020; Paerl, Otten, and Kudela 2018; Paerl et al. 2019; Ibelings et al. 2016), and frameworks for prioritizing and implementing measures for basin-scale load reduction exist (Chorus and Welker 2021). However, sufficient nutrient reduction will require large-scale social change over many years to generations (Jarvie et al. 2013) and may not be sufficient to reverse the rising trend of HCBs. Nutrient control is further complicated by a changing climate that is warming water bodies and promoting more extreme hydrologic events, resulting in a change to how and when nutrients are delivered to and remain in a water body (Paerl et al. 2016). Strategies targeting in situ control and mitigation of HCBs are thus also needed. Further, increasing the effectiveness and predictability of solutions that control and mitigate HCBs where they occur, rather than treating drinking water downstream, is essential to reducing the impacts of HCBs on recreation, fishing, aesthetic values, livestock, and ecosystem structure and function. Relative to the rapid advancements in technology for remote detection of HCBs in large water bodies over the past decade (Cao et al. 2023, 2024; Fang et al. 2022), advancement of in situ control and mitigation of HCBs has been slow.

The portfolio of in situ control and mitigation techniques available is diverse (Burford et al. 2019; Kibuye, Zamyadi, and Wert 2021a, 2021b; Sukenik and Kaplan 2021, Figure 1). Techniques range from experimental to well established, with the potential for unintended consequences. Control and mitigation techniques are also subject to a complicated and poorly-documented record of effectiveness across different limnological settings. As a result, water bodies are regularly treated in a trial-and-error approach for multiple years, at astonishing costs, before an effective solution is identified. This current scenario is likely the result of a number of factors, including: (a) the lack of a policy for establishing responsibility for HCBs and their management; (b) failure of some techniques in some settings, likely a result of undersized systems and/or lack of fit of the technique to the relevant biogeochemical and ecological processes; (c) the experimental nature of some techniques with limited performance data and/or application to limited limnological conditions (e.g., small lakes); (d) the complexity of HCB ecology that produces scientific uncertainty and translates to management uncertainty; and (e) the high costs associated with many of the techniques. Identifying techniques that can effectively control and mitigate HCBs in reservoirs, lakes, and rivers, at reasonable costs and without generating unintended negative consequences, is a complicated task that is dependent on local and regional limnological and political conditions.

Existing literature has tackled HCB control from focused perspectives. Two recent reviews (Summers and Ryder 2023; Tammeorg et al. 2024) summarized HCB control and mitigation techniques focused on removal and utilization of excess nutrients in lakes. Controlling nutrient loading is critical to lake management in some water bodies. However, many sites require HCB control and mitigation techniques that are not necessarily on nutrient control (Matthijs et al. 2016) and/or are used in combination with nutrient management (Lürling and Mucci 2020). A common example of this scenario occurs when a drinking water source may require a short-term solution to prevent an HCB event, but there may not be enough funding, time, responsibility, or resources to implement an effective nutrient control strategy at the basin scale (Chorus and Welker 2021; Lürling and Mucci 2020). The literature has provided summaries of where practices can be effective, though analysis of the factors contributing to the feasibility and effectiveness tend to emphasize individual techniques (Visser et al. 2016; Cooke et al. 2005), and are described as a narrative (Stroom and Kardinaal 2016; Kibuye, Zamyadi, and Wert 2021a, 2021b), rather than being articulated in a structured framework that allows synthesis and comparison of multiple techniques.

The objective of this review is to provide a framework to support scientists and practitioners in identifying appropriate control and mitigation techniques, and their limitations, considering physical, ecological, and socioeconomic factors. We present this framework as a decision tree that can be used by practitioners to narrow the options available to treat HCBs within a specific water body. Case studies are provided to demonstrate how the framework can be used to determine appropriate techniques for different types of water bodies. Synthesis additionally highlights areas where further attention is needed in the science and engineering of HCB control and mitigation. This work builds upon prior work (Burch, Brookes, and Chorus 2021; Ibelings et al. 2016). Ibelings et al. (2016) introduced a matrix of treatment techniques mapped against cyanobacteria taxa and high-level descriptors of lake conditions (e.g., trophic status, mixing type, retention times). We expand upon that schematic by adding more recent literature and techniques, adding limnological and practical factors and constraints (e.g., regulatory, size, cost) that are considered as decisions points by practitioners, and summarizing this complex decision pathway in a simple, structured decision tree. Burch, Brookes, and Chorus (2021) presented checklists of questions to consider for evaluating the prospects for success of a technique at a site. We expand upon these checklists by providing higher-level assessments that direct users to necessary data for making decisions and allow the user to eliminate practices that are not likely to be feasible and effective early in the design phase. We organize the decision process in an easy-to-apply format, and include additional techniques and site constraints.

Finally, in the interest of brevity, we use “control” to refer to the impact or outcome of a treatment in the remainder of this text, though this term was selected with some hesitation. The levels of treatment for HCBs have been discussed in the literature (Ibelings et al. 2016), with control referring to the *elimination* of the risk and nuisance of HCBs, and mitigation referring to the *reduction* of risk and nuisance of HCBs. The term “control” creates some problematic perceptions regarding certainty in outcomes and treatment success, which is unrealistic in many instances, as many prior case studies have shown. Thus, we encourage users to use the term “control” with caution when communicating about these techniques, and to provide adequate caveats regarding the level of confidence in their ability to completely eliminate a HCB.

## 2 | Methods

### 2.1 | Synthesis of Literature

We conducted a comprehensive review of peer-reviewed, agency, and non-governmental organization documents on HCB control techniques, with an emphasis on the limnological conditions under which techniques are appropriate, and case studies where data have confirmed measurable performance. The literature review search took a snowball approach, starting with generic search terms (e.g., “harmful algal bloom mitigation,” “harmful algal bloom control”) that were narrowed as gaps in the retrieved documents were identified (e.g., “harmful algal bloom control mixing,” “harmful algal bloom control light manipulation”). We also collected and reviewed relevant references cited in documents originally acquired through academic search engines. Summaries of reviewed techniques are provided as Supporting Information and Tables 1 and 2 (chemical and physical controls, respectively). From this literature review, we identified factors and techniques to inform development of a national survey on HCB control.

### 2.2 | National Survey

A national survey was distributed to membership of the North American Lake Management Society and state lake manager email distribution lists. Survey questions sought to identify: (a) which HCB techniques were considered common or uncommon, (b) which HCB techniques were considered established or experimental, (c) the most common limnological factors contributing to decisions about control techniques, and (d) if and how the responses varied geographically. Additionally, two open-ended questions sought input on key challenges associated with HCB control. Based on the literature synthesis, factors impacting effectiveness of control strategies were identified and compiled in a list. Techniques included in the survey (and Tables 1 and 2) were based on those listed in the peer-reviewed and gray literature (ITRC 2020; Kibuye, Zamyadi, and Wert 2021a, 2021b). Chemical techniques were classified into three groups (metals-based, oxidants, herbicides) based on (Jančula and Maršálek 2011), where “oxidants” refers to chemicals (e.g., hydrogen peroxide) that inactivate bacteria by generating reactive oxygen species when exposed to light. Readers will note some discrepancies between the labelling of techniques in the survey, Tables 1 and 2, and the decision tree (Figure 5). Because the survey was conducted early in this analysis, the control summaries (Tables 1 and 2) and the decision tree (Figure 5) reflect a more comprehensive perspective on the literature and feedback from respondents, and thus should be considered a more mature organization of the techniques.

We received 105 individual responses, though not every respondent completed every question. Fifty-five percent of respondents were practitioners implementing HCBs control techniques and water quality managers, 23% identified as researchers, 5% identified as having a regulatory role, and 18% reported as having an “other work role related to HCBs.” Survey questions are included as Supporting Information. We only considered responses from respondents located and working in the United States. Results were summarized as proportional and ranked responses, and no statistical analyses were conducted.

### 2.3 | Decision Tree

The goal for developing the HCB control decision tree was to produce a tool for guiding users through the questions and data necessary to narrow the range of appropriate HCB solutions. The decision tree is composed of branches that guide the user through questions about water body and project characteristics, with the terminus of the decision tree being a suite of relevant control techniques. Development of the decision tree was informed by literature review, results from the survey, discussions with practitioners, and our own expertise and experience in HCB limnology and management. The design was iterative, emphasizing limnological processes and contextual details for each technique. The results collectively were interpreted to produce a decision tree with three proposed branches. We verified the validity and applicability of the decision tree design using case studies, five of which are presented herein.

## 3 | Results

### 3.1 | Synthesis of Literature

The Supporting Information: SI includes a thorough review of literature on the HCB techniques identified in Tables 1, 2, and 3. The list is comprehensive, but not completely exhaustive, as some less common and more complex techniques (e.g., modified reservoir operations) were not included. For each practice, the SI synthesis includes a summary of the control mechanisms, limnological conditions under which they are likely to be most effective, unintended environmental consequences (as applicable), and available information on cost and effort. If established limnological thresholds (e.g., depth, cell density, etc.) for appropriateness and effectiveness were found, those are reported in the table and/or summarized in the text.

### 3.2 | Survey Results: National Trends in HCB Techniques

#### 3.2.1 | Factors Contributing to Selecting HCB Techniques

From a list of 21 possible factors that may influence the selection of an HCB technique, respondents ranked: (1) the source of nutrients (internal vs. external); (2) technique cost; (3) cyanobacterial community composition; (4) cyanobacterial cell density; and (5) baseline nutrient load as the top five. Lake (control area) size, water body use and vulnerability, nutrient of concern, dissolved oxygen, and stratification status were among secondary factors.

#### 3.2.2 | Common and Uncommon Techniques

**FIGURE 2 |:**
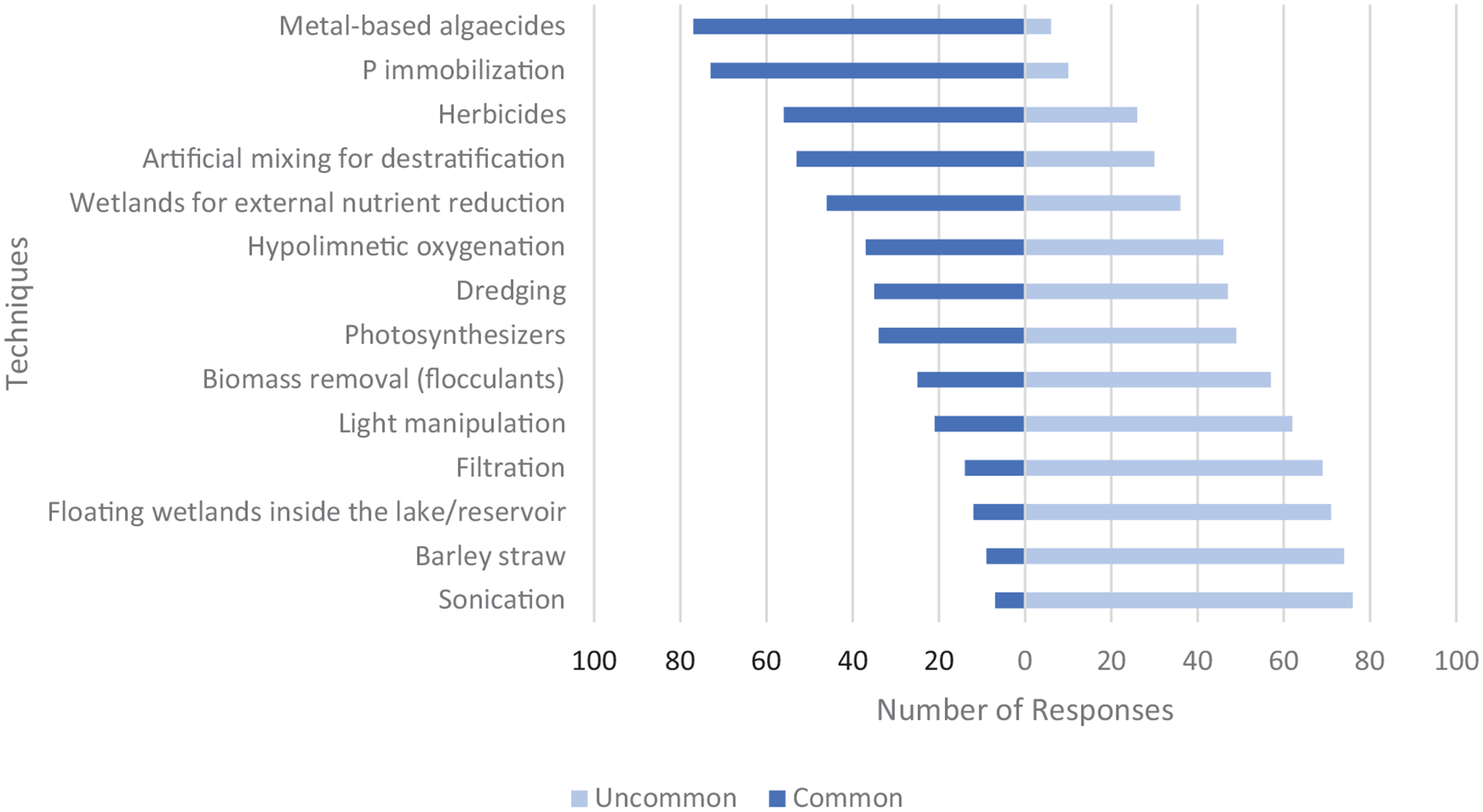
Responses to question: Identify if the following techniques are common or uncommon. Note that we labeled oxidants as “photosynthesizers” in the original survey, consistent with Jančula and Maršálek (2011), referring to the chemicals (e.g., hydrogen peroxide) that inactivate bacteria by generating reactive oxygen species when exposed to light. We subsequently relabeled these chemicals as “oxidants” throughout the paper because this label is more widely recognizable and better reflects their function, but retain the term photosynthesizers here to reflect the questions as presented in the survey.

Nationally, chemical techniques were reported as the three most commonly-applied techniques to mitigate HCBs (Figure 2). Metal-based algaecides, despite being prohibited in some states and applications, are the most frequently used at the national scale, followed by phosphorus (P) immobilization and herbicides. Beyond these chemical approaches, destratification and external nutrient controls were also reported as common.

#### 3.2.3 | Established and Experimental Techniques

**FIGURE 3 |:**
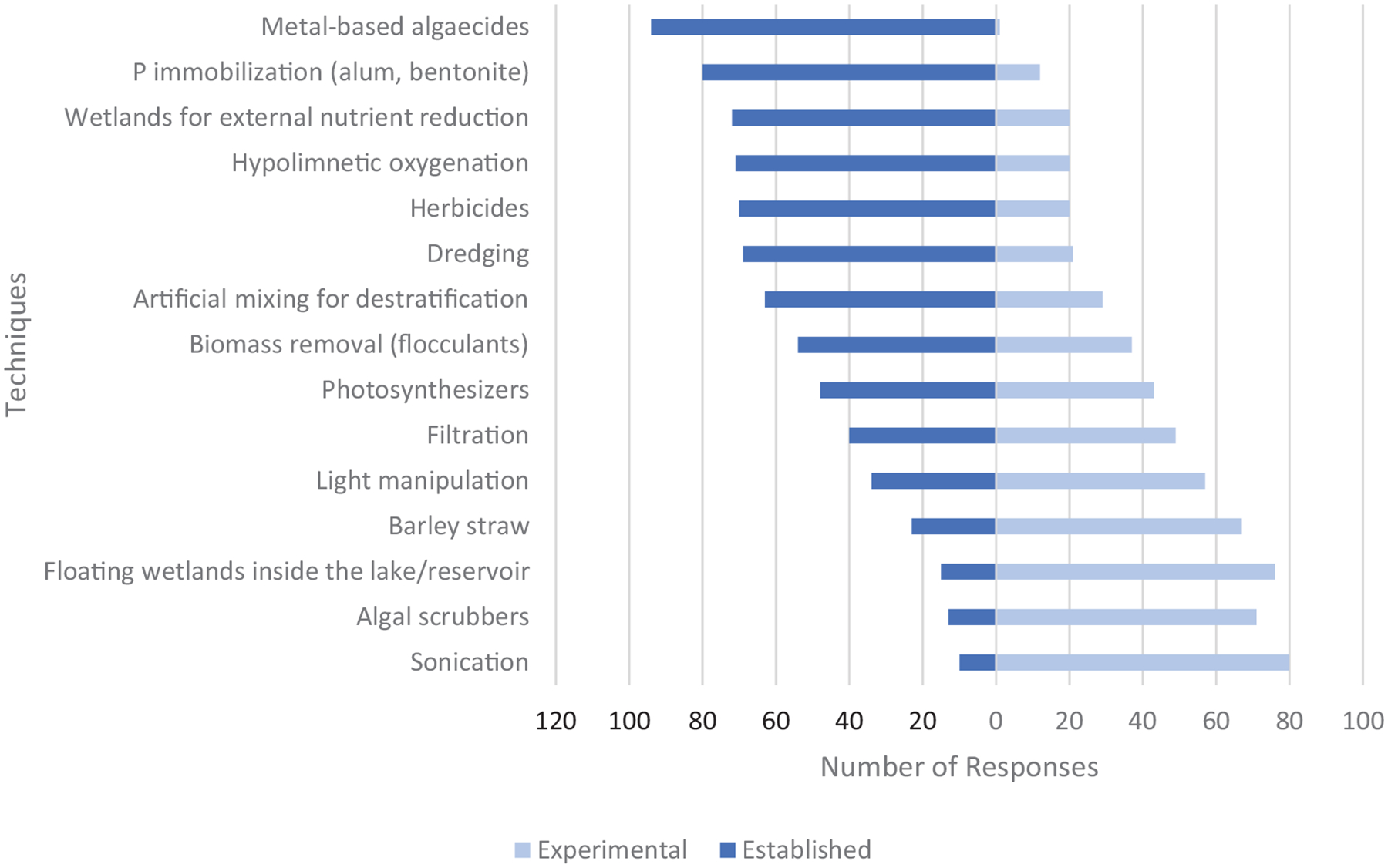
Responses to question: Identify if the following techniques are experimental or established. See note under Figure 2 caption regarding the use of the term photosynthesizers.

Generally, respondents indicated that techniques considered most common were also those considered most established (Figure 3). Conversely, less common techniques tended to be considered experimental by respondents. A few exceptions were reported as techniques considered established but less common, including hypolimnetic oxygenation, destratification, and dredging. Chemical techniques were considered both common and established.

#### 3.2.4 | Geographic Patterns in HCB Control Techniques

**FIGURE 4 |:**
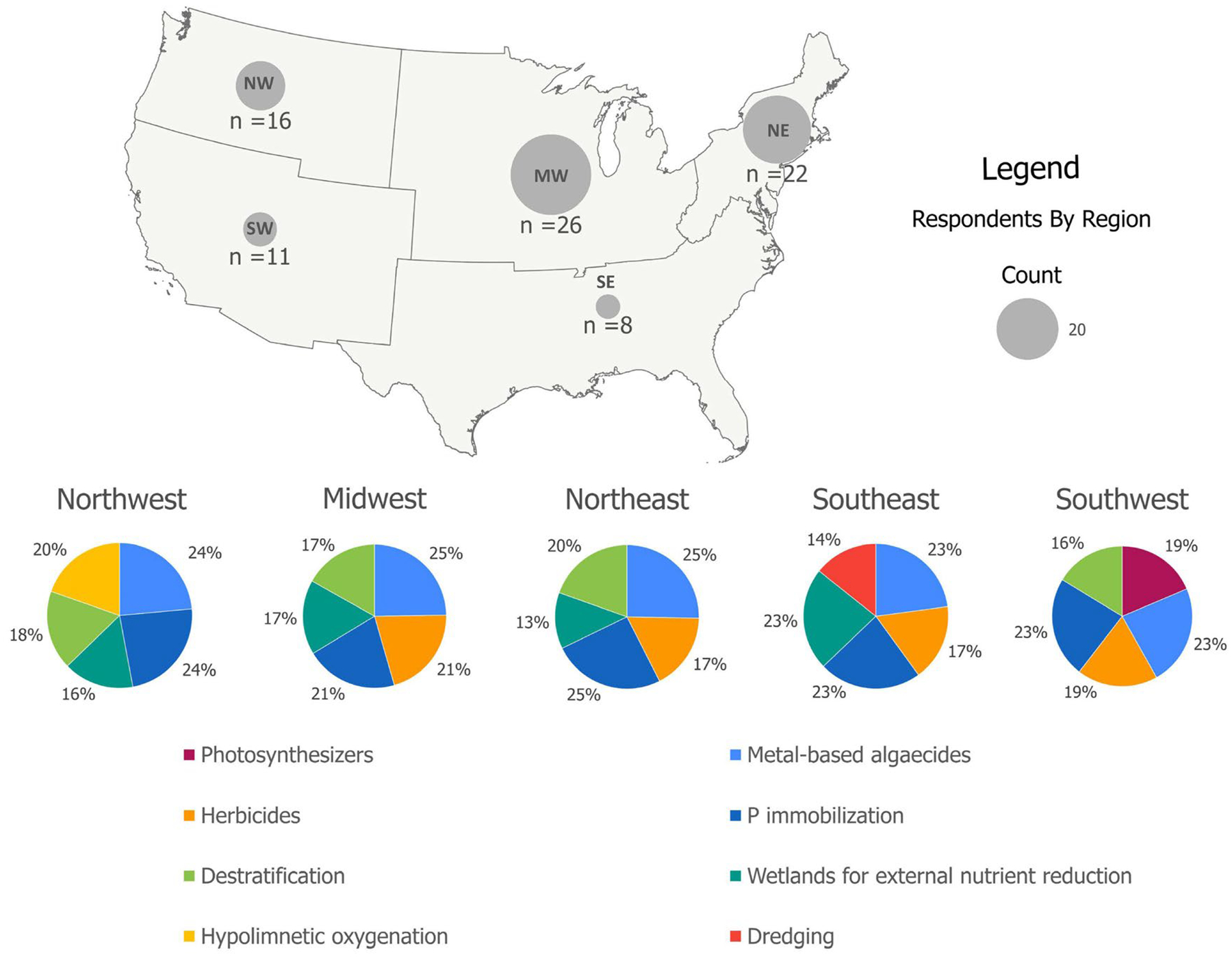
Regional variation in the rankings of the top five most common techniques. See note under Figure 2 caption regarding the use of the term photosynthesizers.

Some techniques are common across all regions, such as metal-based algaecides, herbicides, P immobilization, and wetland establishment/restoration for external nutrient control (Figure 4). Although the number of respondents were limited in some regions (e.g., the Southwest and Southeast), discernible patterns emerged to suggest region-specific differences. For example, dredging was only reported as common in the Southeast. Hypolimnetic oxygenation was only reported as common in the Northwest. Oxidants were only reported as common in the Southwest. In contrast, the eight respondents from the Southeast reported that destratification is uncommon, likely due to a predominance of lakes characterized by more shallow and mixed conditions. Herbicides were reported as uncommon in the Northwest, possibly as a result of strict water quality regulations. These geographic differences illustrate how local geology and hydroclimatic conditions, water infrastructure, and policies and regulation can drive factors promoting or inhibiting a technique. Nevertheless, there are limitations to this survey and a more extensive survey would be needed to fully elucidate how various techniques are utilized in different parts of the USA.

#### 3.2.5 | Barriers to HCB Control

Two open-ended questions highlighted several high-level barriers to mitigating HCBs. Most insightful was the question: *“what single thing would be most useful but is not typically available?”*

The most common response was funding, highlighting the need to connect various partners for an integrated and comprehensive approach to water quality management. Comments reflected a need to prioritize funding on building capacity and implementation of long-term solutions for prevention and remediation. For example, “Biggest thing is funding for AGENCIES, not just universities and researchers, for conducting pilot projects and supporting long-term, high impact solutions.” We need “Less attention to monitoring, detection and management methods that only provide temporary relief. More attention to [complete] remediation and prevention.” Several comments identified the need for policy and funding to address legacy phosphorus and addressing upstream nutrient loads.

Collection and dissemination of baseline and post-control data was the second most-commonly identified barrier. Collection and timely dissemination of watershed and in-lake data are needed to support both planning and analysis of effectiveness. For prevention and control planning, these long-term data needs include “Robust datasets containing multiple years of lake water quality and phytoplankton community composition, watershed hydrology and water quality.” From a control perspective, practitioners need “Easy and reliable access to information regarding successful control strategies in other lakes,” as well as “timely monitoring” immediately prior to and during an HCB. Several responses related to the need for published results and/or basic data on effectiveness of specific practices (i.e., copper and other herbicides, destratification) that were identified as common by the respondents. These issues are summarized in the following comment: we need “more knowledge. Understanding each water body with its nutrient baseline, water budget and inputs can really put you in a better position to ‘win’. Enough staff/technology to perform frequent monitoring to catch blooms early is also a big factor.”

Six comments identified policies and philosophies for managing HCBs as being an issue. Problematic philosophies were identified as being short-term and local. “I wish there were better regulatory mechanisms to control non-point source pollution. Also, I understand that we need short term fixed [sic], but parallel efforts to control nutrient loads into water bodies should not be neglected.” Broadly, respondents characterized the algaecides and other techniques causing direct cyanobacteria mortality as short-term control that fails to address the underlying drivers of HCBs. Several respondents noted that the need to address the source of HCBs as the primary route to decrease the scale, magnitude, and frequency of HCB blooms over the long term. Comments highlighted how culture can be driven by regulatory agencies, noting how “Permitting often is major roadblock to effectively treating cyanobacteria” and that “Forward, strategic thinking government officials” are something missing from the current practice of HCB prevention and control.

Other key factors related to support tools, such as more accurate and rapid laboratory results and predictive models, particularly for upstream nutrient loads, in-lake cyanobacterial dynamics, and toxin production.

### 3.3 | Decision Tree for Selecting HCB Controls

The decision tree is intended as a framework for applying information on limnological and project conditions to help a user narrow the range of appropriate HCB solutions for a specific site. The branches of the tree were developed iteratively, based on results of the literature review and practitioner survey. The decision tree should not be used alone, but instead should be considered in combination with the SI, which provides detailed content on the appropriate application of a technique, summaries of evaluative studies, and costs, where available.

#### Branch 1: Nutrient source (internal vs external).

The origin of nutrients within a waterbody has implications for restoration and control. External nutrient loading refers to the process by which nutrients enter a waterbody from the watershed via erosion/leaching or human sources (e.g., wastewater and agrichemicals) as well as the airshed (e.g., combustion of fossil fuels, agricultural emissions and dust). This external load is then available for assimilation and uptake by phytoplankton and other organisms. Controlling external nutrient loads is typically the first step in improving water quality, but often must be addressed at a regulatory level.

Even if external nutrient loading is suppressed, eutrophication and phytoplankton blooms may continue for some time due to internal nutrient cycling from “legacy” loading of nutrients contained within the sediments. Internally-sourced nutrients are cycled within and released from the sediment (Janssen et al. 2017; Lepori and Roberts 2017; Paytan et al. 2017), and are available for uptake and assimilation by phytoplankton, benthic algae, and other organisms. This is particularly true for P, which cannot be reduced to gaseous and biologically-inert forms. Sediment P release can equal or exceed external P loading in some lakes (Nürnberg and LaZerte 2016; Søndergaard, Bjerring, and Jeppesen 2013; Wu et al. 2017). Internal loading is frequently implicated in continued eutrophication and delayed lake recovery even after decreases in external P loading are achieved (Phillips et al. 2005; Søndergaard, Jensen, and Jeppesen 2005; Søndergaard, Bjerring, and Jeppesen 2013; Watson et al. 2016). Generally, there are more removal pathways for internally-loaded N than P, due to the inherent differences in N and P forms and cycles.

Nutrient source informs the location of, and key mechanisms underlying, appropriate HCB techniques, and therefore understanding these dynamics is a critical first step in effectively managing HCBs. We found in our iterative development process for the decision tree that using nutrient source as the top branch sorted the most techniques initially, making subsequent branches more intuitive. Furthermore, in waterbodies with external nutrient loads, decreasing this load will be the first step necessary in managing HCBs. Once the external load is controlled, other techniques may be appropriate and necessary.

Many systems will have both external and internal nutrient loads, which will require control strategies on both branches of the decision tree. However, external nutrient load will always need to be addressed, as reducing internal nutrient loads alone will not be a sustainable solution if external loads are not addressed. These conditions represent systems where multiple practices are likely needed, which the decision tree forces users to directly confront and consider.

#### Branch 2: Control area size and cost.

The practitioner survey indicated lake size (control area) and cost as the most important factors practitioners use to inform HCB control strategies.

No quantitative thresholds for treatment area or cost have been identified for HCB control. The Interstate Technology Regulatory Council (ITRC) defines “small” lakes as those under 2.4 km^2^ (600 acres) in surface area (Cael, Heathcote, and Seekell 2017), but this designation is based on the distribution of lake sizes globally and therefore is not particularly relevant to questions of effort and cost for HCB control. Instead, this branch separates techniques for which there is either a small control area or a large budget from those for which there is a large control area or a small budget. For example, P immobilization and/or dredging are most appropriate for small control areas or when there is sufficient budget available for HCB control activities since these are relatively expensive techniques. Specific size and cost considerations are likely to vary widely across techniques, management entities, funding source (public or private), and local context, but almost always inform decisions around HCB techniques.

#### Branch 3: Connection to vulnerable ecosystem or regulated receiving waters.

The appropriateness of a control strategy will likely depend on the human and ecological uses for a site. These uses may include drinking water, recreation or spiritual use of the water body, subsistence harvest, and/or presence of vulnerable species protected by state or federal regulations such as the Clean Water Act and/or Endangered Species Act. Some techniques may have unintended negative ecosystem implications that are tolerable in waterbodies managed primarily for recreation, but would not be appropriate for waterbodies containing organisms or other resources that could be negatively impacted by a treatment. This ecological and social context is critical when considering which HCB techniques are appropriate for a given waterbody. Thus, we identified this branch as a key decision point via our own experiences with identifying appropriate HCBs in the systems we manage and work in.

The decision tree is intended to guide users through the three branches, with the terminus of the decision tree being a suite of relevant control/remediation options. Although the decision tree offers a focused list of options based on water body conditions, there will be circumstances in which suggested techniques are deemed infeasible after further investigation. For instance, barley straw may arise as a relevant technique after working through the decision tree, but if dissolved oxygen concentrations in the water body of interest are low, this technique is unlikely to be effective. As such, remediation techniques suggested here should be viewed as a narrowed list from which to begin focused investigation. To assist with further investigation, we include summaries of featured techniques (including specific information pertaining to limnological conditions) in Tables 1 and 2, and the SI. Additionally, more than one condition may apply at a particular site (e.g., where both internal and external nutrient loading are a concern). In this case, the authors encourage users to explore control options available at the terminus of both decision tree branches. Further, taxa-specific cyanobacteria traits may influence the suitability of a technique for a local system, and the user is encouraged to review the SI for a summary of relevant traits and evaluations for specific taxa.

The decision tree is not intended to be quantitative, a deliberate choice because absolute thresholds for cost, nutrient ratios, and ecosystem vulnerability are not universal. There is no single system represented in this tree, but instead it is intended to be generalizable to all systems based on knowledge of local site conditions.

Finally, practices are not always used individually, and the decision tree is intended to provide a suite of potential techniques, some of which may be used simultaneously for best effect. As reflected in the comments of one survey respondent, “I think most of these control measures should be used in combination not just as the sole control.”

### 3.4 | Case Study Applications of the Decision Tree

We demonstrate the use, strengths, and limitations of the decision tree with five case studies. These water bodies were selected to represent diverse ecological and management conditions, ranging from small to large surface areas, varied hydrogeo-graphic conditions and connection to vulnerable ecosystems, and located along the continuum of headwaters to estuaries.

#### 3.4.1 | Upper Klamath Lake, Oregon, USA

Upper Klamath Lake is a large (approximately 251 km^2^ surface area at full pool), shallow, polymictic, and hypereutrophic lake in southern Oregon, USA. Upper Klamath Lake is the primary habitat for endemic and critically endangered Lost River and shortnose suckers; supports a blue-ribbon redband rainbow trout fishery; is an important feeding and resting area for migratory waterfowl using the Pacific Flyway; and is central to the cultural, subsistence, and economic resources of the Federally-recognized Klamath Tribes. Upper Klamath Lake was historically fringed by extensive semi-permanent and permanent emergent peat wetlands that provided both physical habitat and important controls on P and water quality. Compounding the loss of the majority of these wetlands, substantial changes within the watershed, including channelization, construction of dikes/levees, unmanaged riparian grazing, timber harvest, and water diversions to support agricultural operations, have resulted in increased P load to the lake since the early 1900s (Eilers et al. 2004; ODEQ 2002; Platt Bradbury, Colman, and Reynolds 2004).

Extensive and often toxic cyanobacterial blooms proliferate in Upper Klamath Lake each summer, creating challenging water quality conditions for aquatic organisms, and affecting recreational and aesthetic values. Poor water quality is one of several factors implicated in the decline of endangered suckers (USFWS 2012). Multiple studies (e.g., ODEQ 2002; Wherry and Wood 2018) indicate that limiting the total P load from the watershed is the key to minimizing these blooms and improving water quality in the lake. Furthermore, total P entering Upper Klamath Lake from the watershed each winter is tied directly to that available to cyanobacterial blooms in early summer (Walker and Kann 2020). There is a Total Maximum Daily Load for external P load to Upper Klamath Lake (ODEQ 2002).

**FIGURE 5 |:**
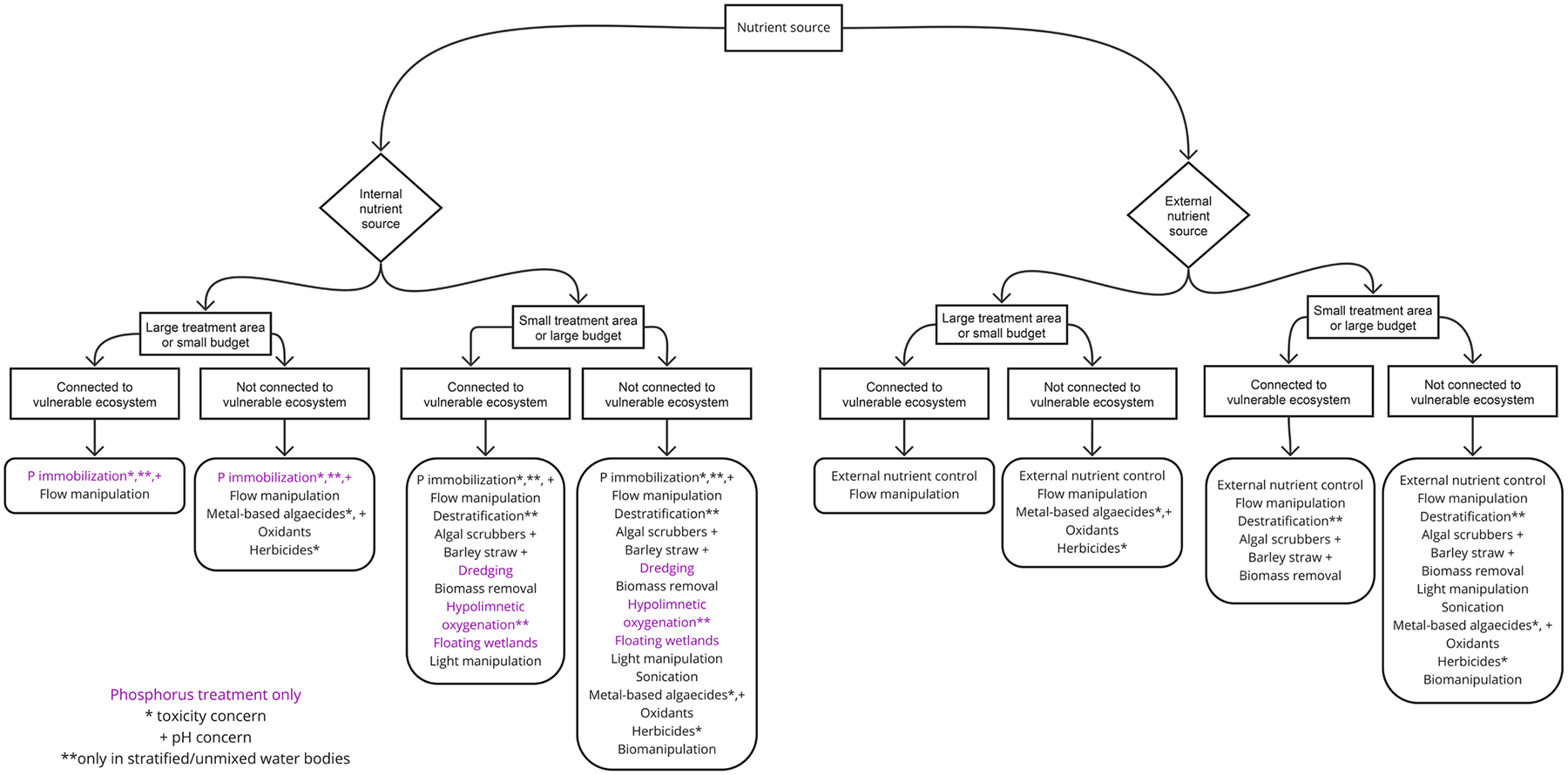
Decision tree for selecting HCB controls based on local conditions.

Using the Decision Tree (Figure 5), Tables 1 and 2, and the SI to inform HCB control/remediation techniques for Upper Klamath Lake:

1. External loading is the primary source of P to Upper Klamath Lake (Walker and Kann 2020).
2. Upper Klamath Lake is a large lake (surface area of 251 km^2^), meaning it is a large control area. There has been past consideration for control in specific bays or other areas of the lake, but currents (including a gyre) within the lake, and the ability of cyanobacteria to rapidly repopulate, make treating smaller areas less likely to be effective.
3. Upper Klamath Lake is the primary remaining habitat for critically endangered Lost River and shortnose suckers, and supports subsistence resources for The Klamath Tribes. It is therefore considered a vulnerable ecosystem.
4. Given the factors above, the suggested techniques for Upper Klamath Lake include *external nutrient control* and *flow manipulation*.
5. Further examining these techniques in Tables 1 and 2 and the SI we conclude the following:
  - *External nutrient control* is a feasible technique considering that numerous studies indicate external P load from the Upper Klamath Lake watershed drives cyanobacterial blooms and resulting suboptimal water quality conditions in the lake.
  - Upper Klamath Lake is relatively shallow with substantial external nutrient load, meaning vertical mixing is unlikely to be effective. Additionally, the number of mixers necessary to achieve sufficient coverage across the lake surface is likely cost prohibitive given the very large lake surface area and currents distributing cyanobacteria throughout. Strong year-round regulatory control of the timing and scale of Upper Klamath Lake outflows (balancing the needs of several endangered fish species and irrigation) renders hydraulic flushing largely infeasible. Thus, *flow manipulation* is likely not a feasible technique.

In conclusion, the decision tree, Tables 1 and 2, and the SI indicate *external nutrient control* is the best option for remediating cyanobacterial blooms in Upper Klamath Lake. This technique includes a number of actions such as wetland, riparian, and floodplain restoration, and irrigation management (specifically to decrease the P load associated with irrigation tailwater returns to the lake and tributaries) (The Upper Klamath Basin Watershed Action Plan Team 2021). There has been slow, but steady, progress towards implementing restoration sufficient to achieve water quality objectives. Challenges to controlling external P load to Upper Klamath Lake include the size of the watershed (nearly 5000 km^2^), landscape modification therein, and the nature of property ownership (largely private).

#### 3.4.2 | Neuse River and Estuary, North Carolina, USA

The Neuse River estuary (NRE) in North Carolina is 64 km long, 10 km wide at its mouth, covering 1500 km^2^ acres of estuary. It is home to many species, including oysters, birds, coastal game fish, dolphins, alligators, sharks, and manatees. The Neuse River is also home to many endangered species, including the Carolina madtom, piping plover, and loggerhead turtle. Since the late 1970s, approximately 120 km of the river and estuary has suffered from nutrient-driven HCBs responsible for major fish die-offs, enhanced bottom water hypoxia, and degradation of benthic and planktonic habitats (Paerl et al. 1995; Paerl and Piehler 2008). The NRE originates in the Piedmont of North Carolina and empties into the Pamlico Sound, part of the USA’s second largest estuarine complex, the Albemarle-Pamlico Sound System. The downstream coastal plain segment largely drains agricultural lands, including row-crop and intensive poultry and swine operations. The basin supplies water to the population of nearly 1.4 million. Over the next 25 years, the human population in the watershed is expected to grow by 53%, while poultry operations are expected to increase by 39%. Approximately 80% of nitrogen (N) and P loading to the NRE is of non-point source origin, heavily dominated by agriculture (> 50%), while ~20% is from point sources, reflecting wastewater and industrial discharges. Agriculturally, nearly all nutrients applied were from commercial fertilizer and animal waste. Phosphorus input reductions, including advanced wastewater control, a P detergent ban, and construction of riparian buffers around agricultural lands were undertaken in the late 1990s to decrease external P loading (Paerl et al. 2004). However, parallel emphasis on external N input reductions were not pursued at that time, despite the fact that the downstream NRE exhibited N-stimulated phytoplankton blooms, accompanied by extensive hypoxia and fish die-offs (Paerl et al. 1995; Paerl and Piehler 2008). The HCBs occur in the oligohaline segment of the NRE, producing chlorophyll a (Chl a) concentrations at times that exceed 100 μg/L (Paerl et al. 2006), in excess of the “acceptable” North Carolina Chl a standard of 40 μg/L.

Using the Decision Tree and narrative to inform cyanobacteria control techniques for the Neuse River and estuary:

1. External nutrient source: Key sources of nutrients clearly originate from upstream urban and agricultural land uses.
2. Large control area: The reach of the lower river and estuary affected by the blooms is ~120 km.
3. Vulnerable ecosystem: The NRE contains numerous shell-fish and finfish species that are commercially and recreationally caught and consumed. In addition, the state of North Carolina prohibits the application of toxics for algacidal control, except in small systems (such as golf course ponds) that are not hydrologically connected and subject to direct human exposure and consumption.

Given the factors above, the suggested techniques for NRE include *external nutrient control and flow manipulation*.

- *External nutrient control* was ultimately implemented with varied results, as discussed below.
- *Flow manipulation* was not possible in this river setting due to the large size of the system.

In 1999, NC Department of Natural Resources and the US EPA implemented a N-driven Total Maximum Daily Load (TMDL), which mandates a 30% reduction in total N inputs. Recovery in the downstream NRE has been uneven, with widespread decreases in nitrate concentrations but parallel increases in ammonium and organic N concentrations (Gabriel et al. 2018; Lebo, Paerl, and Peierls 2012).

As of 2009 and 2018, the North Carolina state standard for Chl a (40 μg/L) was still in exceedance in the Neuse River Estuary TMDL (Keith 2014; Paerl, Crosswell, et al. 2018; Paerl et al. 2004; Paerl, Otten, and Kudela 2018). A complicating factor has been a rise in tropical cyclone frequency and intensity since the mid-1990s, causing more extensive watershed flooding and increases in pulse external loading of nutrients, especially organic N forms throughout the riverine-estuarine continuum (Paerl, Otten, and Kudela 2018). Further decreases in both N and P external inputs are needed, including extending edge of field agricultural riparian buffers to intercept stormwater runoff in the rapidly urbanizing areas of the catchment. While there has been progress made in reducing nitrate-rich chemical fertilizer applications, more aggressive reductions in agricultural fertilizer applications and organic and ammonium-rich animal waste release (through surface runoff, groundwater discharge and atmospheric emissions), which account for > 50% of total watershed/airshed loads, are needed (Lebo, Paerl, and Peierls 2012) The current recommendations are to hold the line on P inputs while decreasing N inputs, below the current 30% TMDL reduction levels. The Neuse freshwater-to-marine continuum reflects a global need for more aggressive decreases in external N and P loads to reverse eutrophication, proliferation of harmful algal blooms, hypoxia and fisheries habitat loss (Paerl et al. 2016).

#### 3.4.3 | Ross Island Lagoon, Oregon, USA

Ross Island Lagoon (RIL) is a deep (40 m), stagnant pool in the middle of the lower Willamette River, a tidally-influenced reach that runs through the heart of downtown Portland, Oregon (Tullos, Fay, and Levenson 2023). The lagoon has a long history of manipulation, beginning in the 1920s when the US Army Corps of Engineers constructed an embankment that shut off downstream flow through what is now RIL. Following the closure of the upstream end, a private gravel operator removed millions of cubic meters of gravel and sand from the lagoon. These activities have contributed to the HCB at RIL, which has been occurring with increasing frequency and intensity, and has led to the listing of the entire lower Willamette River as impaired for cyanobacteria under the TMDL program. Recreational contact advisories have been issued for the last 7 of 10 years, and the lagoon is part of the Lower Columbia Ecologically Significant Unit for winter steelhead and spring Chinook salmon. Given the history of the lagoon, there is no single responsible party. Instead, securing funding and building capacity for analysis and control of the HCB has been led by a local non-profit. Given the very public setting in the middle of a very urban area, substantial effort was invested in outreach with local and state agencies and the public.

Hydrodynamics of the 0.6 km^2^ lagoon are complex, resulting in a system that is not quite a lake and not quite a river. The center of the lagoon is strongly stratified while the shallow margins of the lagoon are wind-mixed. Although the lagoon is connected to the river through a side channel, the river only accesses the lagoon through the diurnal tidal cycle. Although tidally influenced, RIL is not subject to salinity pulses. Instead, the tidal cycle is produced by the mainstem Columbia River, which rises with the tide and prevents the Lower Willamette River from draining (rather than a tidal pulse moving upstream). That tidal cycle does deliver elevated N and P loads to the lagoon from the river, and a pool of nutrients also exists in the anoxic hypolimnion. The tide is also responsible for the export of cyanobacteria produced in the lagoon. In 2023, the recreational advisory and bright green hue extended throughout the lower Willamette River along downtown Portland. Beyond ecological impacts, local partners view the HCB as an environmental justice issue. The river serves as a cooling refuge during the increasingly frequent heat waves in an area with widespread lack of air conditioning among inner-city residents in the Pacific Northwest.

Using the Decision Tree to inform cyanobacteria control techniques for Ross Island Lagoon:

1. External nutrient source: Although internal and external nutrient sources exist, the external nutrient source is considered to control nutrient loads in the lagoon.
2. Large control area: With a surface area of 0.6 km^2^, the control area of RIL is considered small relative to the global distribution of lakes. However, the substantial depth and varied hydrodynamics of the lagoon make some techniques (e.g., barley straw, hydrogen peroxide, dredging) cost prohibitive and/or unlikely to be effective. Thus, the site is considered to be a large treatment area.
3. Vulnerable ecosystem: RIL shallow water habitats identified in the recovery plan for ESA-list Chinook Salmon and steelhead, and the state of Oregon restricts use of some chemical algaecides (e.g., copper, alum) in public waters.

Given the factors above, the suggested techniques for RIL include *external nutrient control and flow manipulation*.

- *External nutrient control* is unlikely to be feasible because upstream sources of nutrients derived from extensive agriculture across the entire Willamette River basin without a strong TMDL program targeting nutrients.
- *Flow manipulation*, via a hydraulic flushing channel, was identified as the favored alternative due to the low energy and carbon costs, the minimal need for ongoing operation and maintenance, and the ecological benefit of restoring connectivity and fish access between the main channel and the lagoon.

As a result of complex hydrodynamics, internal and external nutrient loads, and its location within a vulnerable ecosystem, the favored alternative at RIL is construction of a flushing channel to increase mixing and reduce conditions for phytoplankton production within the lagoon. The proposed channel will be located and positioned to avoid disturbance of capped contaminants. The project is currently undergoing detailed design and cost analysis.

#### 3.4.4 | Anonymous Northern California Lake, USA

A northern California agency operates a small, shallow, well-mixed waterbody that occupies 0.07 km^2^ and stores approximately 300,000 m^3^ of tertiary treated recycled waste water. Maximum operating depth of the waterbody is 14.3 m, but typical operating depths range from 1.5–3.1 m. Stored water can be distributed to numerous recycled water users or discharged to a nearby creek. The advanced-treated, recycled water is used to irrigate urban and agricultural lands, a golf course, local farmlands, vineyards, a park, irrigation and toilet flushing, a high school, and other parcels. Due to the nature of the water source, the waterbody has consistently high concentrations of nutrients, with total N ranging from 3 to 6 mg/L and total P ranging from 0.5 to 2 mg/L.

Microcystin-producing HCBs proliferate in the waterbody each summer, creating poor water quality conditions that can negatively impact those contacting the water and may limit beneficial uses of the water. A routine monitoring program detected microcystins in 2018. Maximum microcystin measurement to date is 2318 μg/L. However, maximum microcystin concentrations in the recycled water distribution system have only reached 2.38 μg/L. To control cyanobacteria and reduce occurrences of microcystin, the municipality conducted a control feasibility evaluation. The following information provides the considerations using the Decision Tree and narrative for this small northern California waterbody:

1. External nutrient source is the primary source of nutrients.
2. With a surface area of 0.07 km^2^, the northern California lake is a small waterbody, meaning it is a small control area.
3. The waterbody that produces tertiary treated recycled water is not in a vulnerable ecosystem. However, the water discharges to irrigation users and in the winter months when cyanobacteria levels are low, the water may be discharged to a nearby creek that is considered a vulnerable area.
4. Given the factors above, the techniques considered to mitigate HCBs in the northern California lake include *external nutrient control, flow manipulation, destratification*, *algal scrubbers*, *barley straw, biomass removal, light manipulation, sonication, metal-based algaecides, oxidants, herbicides, and biomanipulation*.
5. Further examining these techniques in the narrative, we conclude the following:
  - *External nutrient control* is not considered to be a feasible technique due to the source of the lake’s water being recycled water. Any control that would decrease the nutrient supply would be costly or result in recycled water permit violations.
  - *Flow manipulation was* considered feasible for restricting the ability of cyanobacteria to regulate buoyancy in the water column, thus allowing other algal species to compete with cyanobacteria. Mixers were installed and are operated during the HCB season.
  - The lake is shallow and does not stratify, thus *destratification* was not considered feasible in this setting.
  - Given the prolific nature of HCBs in the lake, *biomass removal* is not a feasible technique.
  - A solar array was installed across approximately 0.03 km^2^ of the lake. The purpose of the solar array was to generate power. However, the solar array has also been effective at blocking sunlight to cyanobacteria. Thus, *light manipulation* has been effective in this waterbody.
  - Due to the size and geometry of the lake, as well as the presence of the solar array and tether cables, *sonication* was determined to not be a feasible solution to mitigate cyanobacteria in the lake.
  - The State permit regulating the recycled water disal-lows modifying the chemical character of the input water without approval from regulatory agencies. As such, utilizing *metal-based algaecides*, *oxidants*, or *herbicides* would result in a violation of the permit, and is therefore considered not feasible.
  - *Biomanipulation* was not feasible because biomanipulation is less successful in highly eutrophic lakes with very high nutrient loads due to higher phytoplankton growth rates and because there are more non-edible species, such as colonial or filamentous cyanobacteria.

Implementation of the feasible control methods (artificial mixing and light manipulation) has been successful. There are occasional small HCBs, but microcystins in the recycled water distribution system have remained below the state of California’s “Caution” threshold of 0.8 μg/L. This waterbody is similar to a number of small waterbodies in parks, golf courses, and other urban areas, which tend to be very eutrophic and reflects a scenario of how to manage a waterbody where nutrient reductions were not feasible.

#### 3.4.5 | Lake Trummen, Kronoberg, Sweden

Lake Trummen is a small (surface area of approximately 1 km^2^, mean depth 1.6 m) lake that experienced HCB-related water quality degradation in response to domestic sewage and industrial nutrient inputs during the mid-1900s (Bengtsson et al. 1975; Cronberg 1982). Formerly oligotrophic, Lake Trummen near the town of Växjö was heavily polluted by municipal and industrial wastewater from the mid 1930s until 1958, leading to regular fish die-offs resulting from anoxic conditions and regular HCB blooms. The inflow of wastewater ended in 1958, but the lake did not recover. Preliminary investigations in 1968 and 1969 indicated the documented 20–50 cm of black, loose sediment deposited during the pollution, which contributed most to the high productivity of the lake during the spring and especially the summer (Andersson et al. 1973, Björk and Digerfeldt 1965). Internal loading as a result of sustained historical external P load was the primary source of P feeding the HCBs. Nutrients released from the “black sediment” gave rise to extensive and persistent HCBs, dominated by *Microcystis aeruginosa, Aphanizomenon flos-aquae*, *Anabaena flos-aquae* and *Oscillatoria* sp.

However, while wastewater discharges ended in 1958, uncontrolled external nutrient loads continued until the 1970s. Because both external and internal nutrients were a concern at the time of assessment, we will consider both branches of the decision tree as a decision manager would have in 1970 when restoration actions were undertaken.

Using the decision tree to inform cyanobacteria control techniques for Lake Trummen:

1. Internal and external nutrient source: The discharge of untreated wastewater ended in the 1950s, though uncontrolled external nutrient load continued until the 1970s.
2. Small control area: With a surface area of approximately 1 km^2^ and a mean depth of 1.5 m, the control area of Lake Trummen is relatively small.
3. Vulnerable ecosystem: Lake Trummen is not connected to habitats for any protected species or a source of drinking water, but is used recreationally, and thus is classified as being subject to vulnerable uses.

Given the factors above, a large number of treatment options are potentially feasible based on the decision tree:

- *Destratification* was not a relevant option because the shallow lake was not stratified.
- *Algal scrubbers* were not feasible due to the cool climate and short summers in Sweden.
- *Barley straw* was not yet known as a water quality improvement approach in the 1970s.
- *Biomass removal* was infeasible since the HCBs were present in both the water column and widely distributed in the flocculent sediments.
- *Dredging* was selected as the control technique for this lake in 1970 due to the small lake size, the effectiveness of this technique in decreasing internal loading, and in part due to the lack of other control techniques available in the 1970s.
- *Hypolimnetic oxygenation* was not relevant because the shallow lake was not stratified, and this technology was just emerging as a new practice in the 1970s.
- *Floating wetlands* were not yet known as a water quality improvement technique in the 1970s, and likely would have been ineffective anyway, considering the relatively short growing season.
- *Flow manipulation* was not considered due to lack of water available for flushing the system.

The restoration of Lake Trummen was carried out during 1970 and 1971 by means of (1) a suction-dredging method, whereby nutrient-rich sediment was pumped up into settling ponds on abandoned farms (Björk, Pokorný, and Hauser 2010), to address internal loads, and (2) a strict external nutrient reduction program to address remaining external loads. Suction dredging of the upper meter of sediments during a 2-year period led to substantial decreases in nutrient concentrations and HCBs (Bengtsson et al. 1975; Cronberg 1982; Andersson et al. 1973). Runoff from the settling ponds was treated with aluminum sulfate to immobilize P (Björk, Pokorný, and Hauser 2010).

Reductions in P and N concentrations in the lake were dramatic (> 80% reduction in peak total P), the cyanobacterial blooms were eliminated, and a freshwater mussel was re-established (Björk, Pokorný, and Hauser 2010). The decreased internal loading following dredging observed in the 1970s remains, as a result of both sediment removal and strict reductions in external nutrient loading (Welch and Cooke 1999). In connection with the restoration, the mean biomass of phytoplankton from June until September decreased from 75 to 10 mg/L, mainly due to the lower abundance of HCBs (Andersson et al. 1975). The annual production in 1972 was calculated to be 245 g carbon/m^2^, a decrease of ~30%, relative to that observed in 1969. In post-dredging 1972, a much greater part of the total phytoplankton production was due to small eukaryotic phytoplankton when compared to 1969 (Andersson et al. 1973). Lake water quality was considered high until the early 1990s, when cyanobacteria again began increasing. The lake under-went two periods (1994–2000, 2015–2021) of biomanipulation to remove planktivores (bream). Reducing the planktivores resulted in increases in zooplankton, which increased grazing of phytoplankton (LOVA 2019). The reduction of the planktivores apparently also reduced disturbance of the bottom sediments, thus reducing phosphorus mobilization and macrophyte disturbance.

Today, water quality at Lake Trummen is consistently rated as high, with Chl a values below 20 μg/L since 2016 (https://miljodata.slu.se/MVM/Search). Diatoms and flagellates dominate the plankton community and macrophytes have re-establishment (Växjö 2021), making for a popular recreational resource for fishing, swimming, windsurfing, and other activities.

The Lake Trummen success can largely be attributed to its small, easily manipulatable size, and the ability to effectively target decreases in external nutrient loads from its small (13 km^2^) watershed, following dredging. Further, we note that, in addition to the small lake size, this extreme dredging scenario was possible in part due to a permissive regulatory environment and would likely not be feasible in all small lakes today. Finally, we note that, in using the decision tree, this site was classified as vulnerable due to the recreational use of the lake. Recreational uses may not prohibit some of the current control strategies that are included in the list for sites not subject to vulnerable uses, such as metal-based algaecides, oxidants, herbicides and P immobilization. Recreational use is one point in the decision tree where the user may have to use knowledge of local regulations and site conditions before eliminating some techniques.

## 4 | Conclusions

HCB techniques have a mixed history of success for a variety of reasons, and practitioners report that key barriers to HCB control include lack of funding, particularly for planning and implementing long-term solutions, post-implementation data to support effectiveness evaluations, and policies and philosophies that emphasize short-term solutions. There is a mix of economic, social, and limnological factors that drive selection of HCB control techniques. Nationally, chemical applications appear most common, though dominant techniques vary geographically, likely a result of variation in hydroclimatic conditions and environmental regulations. Nutrient source (internal vs. external), the size of the control area and project budget, and the vulnerability of the ecosystem all play key roles in determining the appropriateness of specific techniques for a given water body. Every water body must be assessed individually to ensure control approaches will be effective and sustainable from both and ecological and cost perspectives (Tammeorg et al. 2024). Case studies demonstrate how these factors play out in the real world.

In the decision tree development process, we encountered a number of data gaps. The following topics warrant further investigation to inform effective control approaches in the future:

1. A number of techniques were deemed “experimental.” Techniques such as algal scrubbers, sonication, and light manipulation require further field-based experiments to determine if investments in such techniques are warranted.
2. Even for “established” techniques (e.g., algaecides, P immobilization), there is limited peer-reviewed documentation providing information on appropriate dosing, or dose frequency. Publicly-available data and a compilation of information on monitored case studies are necessary to establish effectiveness and longevity of various techniques to inform financial and ecological decisions.
3. There is little information evaluating the combination of different lake management techniques. Many water bodies, especially larger lakes where there is more funding available, utilize a combination of techniques to mitigate HCBs. Yet, written assessments of these studies are lacking.
4. Systematic monitoring during HCBs, and with implementation of control techniques, is essential. This information is often lacking, which can make it difficult to determine if a technique is effective. More fundamentally, more information of this nature will help fill critical science gaps around the microbiological and biogeochemical interactions driving HCBs and their senescence.
5. A common theme that emerged in the reviewed literature was that practitioners are unsure how control techniques will respond to climatic changes such as flashier precipitation events, warmer temperatures, and changing biogeochemical processes. Studies evaluating management techniques under various climatic conditions will assist practitioners in understanding how long-term control will be impacted by such changes.

## Supplementary Material

Suppl 1

Suppl 2

## Figures and Tables

**FIGURE 1 |:**
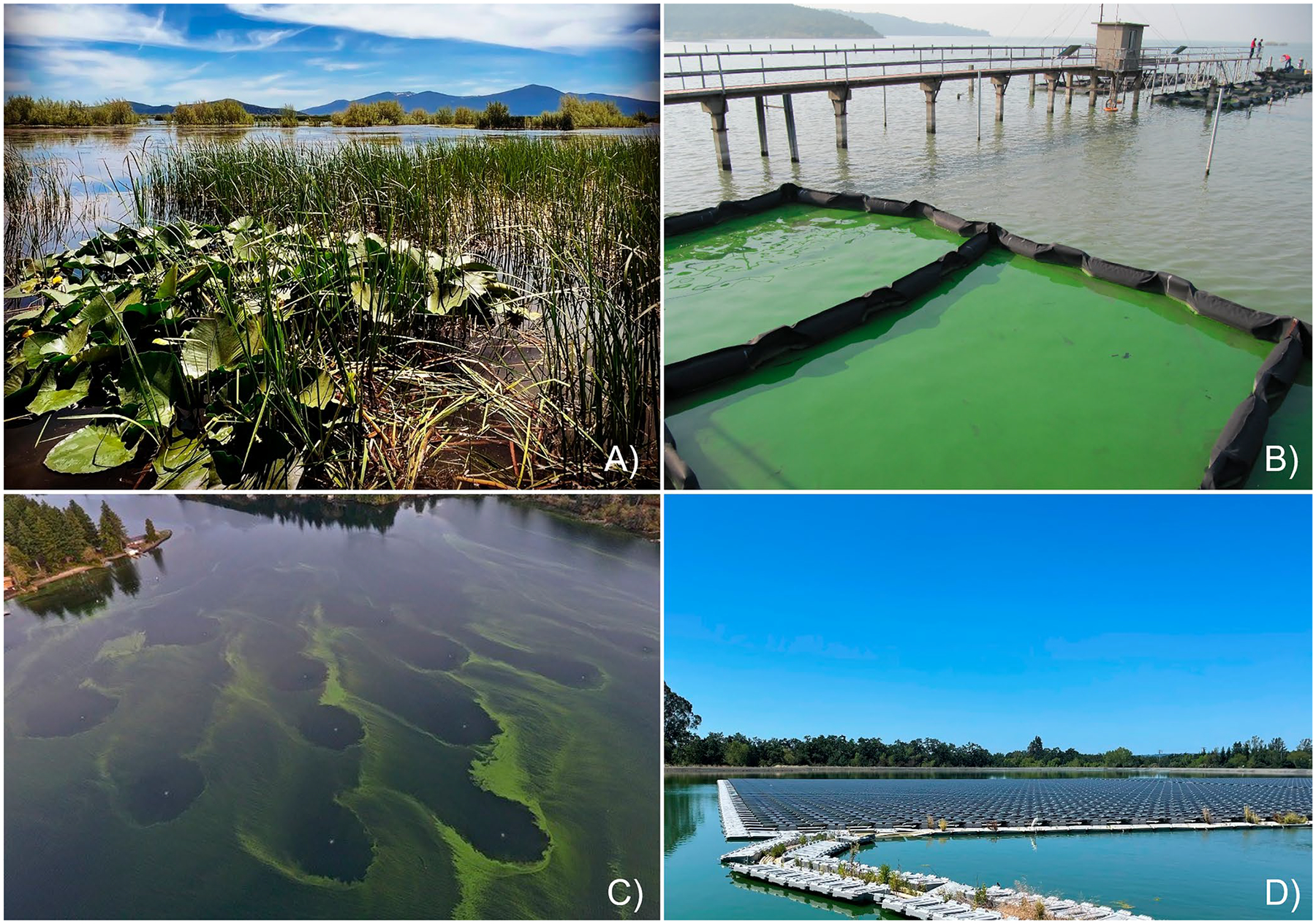
HCB mitigation techniques: (a) Wetland restoration for external nutrient control. *Source:* Megan M. Skinner; (b) Isolation for biomass removal. *Source:* Hans W. Paerl; (c) Diffusion aeration (Josh Cohen); (d) solar array. *Source:* Ellen P. Preece.

**TABLE 1 |:** Summary table of chemical techniques.

Treatment	Target	Experimental or established?	Factor	Effective conditions	References
Herbicides (e.g., Diuron, Endothall)	Cyanobacteria	Established	Cyanobacteria community composition	Effectiveness may depend on if cyanobacteria have gas vesicles vs. no gas vesicles or colonial vs. filamentous.	Lefler et al. (2022)
			Cyanobacteria cell density	Should be used in the early stages of bloom development while cell density is low. Early application enhances effectiveness and reduces toxin release.	Kinley-Baird et al. (2021), Sukenik and Kaplan (2021)
Oxidants (e.g., hydrogen peroxide)	Cyanobacteria	Experimental	Solar irradiance	More effective with higher solar irradiance.	Piel et al. (2019); summarized in Kibuye, Zamyadi, and Wert (2021a)
			Wind-induced mixing	More effective when wind-induced mixing is present.	Barrington, Reichwaldt, and Ghadouani 2013
			Phytoplankton cell density	Should be used in the early stages of bloom development while cell density is low. Early application enhances effectiveness and reduces toxin release.	Kibuye, Zamyadi, and Wert (2021a); Weenink et al. (2021)
			Taxa-specific responses	*Planktothrix* highly sensitive, *Microcystis, Pseudanabaena*, and *Raphidiopsis* moderately sensitive; others untested	Lusty and Gobler, 2020; Sandrini et al. 2020; Weenink et al. 2021; Kibuye, Zamyadi, and Wert 2021a, 2021b
			Dissolved organic matter	More effective when dissolved organic matter concentrations are low.	Paerl and Otten (2013)
Metal Algaecides (e.g., copper-based algaecides)	Cyanobacteria	Established	Solar irradiance	More effective at low solar irradiance	Watercourse Engineering (2013), Greenfield et al. (2014)
			Suspended solids	More effective when suspended solids concentrations are low.	Raman and Cook (1988)
			pH	More effective at pH values 5 to 7	Zeng, Yang, and Wang (2010)
			Water temperature	More effective at water temperatures > 10°C	Raman and Cook (1988)
			Alkalinity	More effective when alkalinity is high.	Kibuye, Zamyadi, and Wert (2021a)
			Primary water body purpose	Most appropriate for water bodies used for drinking water and/or recreation and is not for those with vulnerable species and/or subsistence resources.	Hanson and Stefan (1984); Moore and Winner (1989); Williams et al. (2015); Kibuye, Zamyadi, and Wert (2021a)
			Cyanobacteria cell density	Should be used in the early stages of bloom development while cell density is low. Early application enhances effectiveness and reduces toxin release.	Kibuye, Zamyadi, and Wert (2021a); summarized in Stroom and Kardinaal (2016)
Algal Scrubbers	Cyanobacteria	Established	Topographic complexity in raceways	More effective when raceways include topographic complexity (i.e., more colonization sites).	Adey, Kangas, and Mulbry (2011)
			Growing season length	Most effective in climates with relatively long growing seasons (e.g., Mediterranean and sub-tropical) rather than short growing seasons (e.g., temperate, boreal, and arctic).	Dinkins et al. (2009); Adey, Kangas, and Mulbry (2011)
			Baseline nutrient concentrations	More effective when inflow nutrient concentrations are higher.	Adey, Kangas, and Mulbry (2011)
			pH	More effective at pH > 8	Ferguson, Jenkins, and Eastman (1973)
Phosphorus Immobilization	Nutrients	Established	Residence time	More effective when residence time is long.	Hickey and Gibbs (2009)
			Source of nutrients	More effective in systems with a predominantly internal nutrient source. It is less commonly used in systems with external nutrient loading.	See references in Supporting Information.
			Mixing frequency	More effective when mixing frequency is low.	Hickey and Gibbs (2009)
			Dosing	Effectiveness influenced by dosing, which can be hard to predict due to impact of local characteristics on appropriate dose and frequency; overdosing can impact ecosystem.	Hickey and Gibbs (2009)
Floating Wetlands	Nutrients	Established	Nutrient of concern	More effective in treating nitrogen and less effective for phosphorus.	Stewart (2007), Stewart et al. (2008), Lynch et al. (2015), Spangler et al. (2019)
			Growing season length	Most effective in climates with relatively long growing seasons (e.g., Mediterranean and sub-tropical) rather than short growing seasons (e.g., temperate, boreal, and arctic).	Pavlineri, Skoulikidis, and Tsihrintzis (2017)
			Dissolved oxygen concentrations	Most effective when oxic conditions are maintained and less effective under hypoxic or anoxic conditions.	Stewart (2007), Stewart et al. (2008)
			Baseline nutrient concentrations	More effective in treating nutrients when inflow nutrient concentrations are higher (e.g., wastewater, stormwater).	White et al. (2009); Lynch et al. (2015); Pavlineri, Skoulikidis, and Tsihrintzis (2017); Garcia Chance et al. (2019); Spangler et al. (2019)
Barley Straw	Cyanobacteria	Experimental	Dissolved oxygen concentrations	Barley straw is most effective in oxic conditions.	Pillinger, Cooper, and Ridge (1994)
			pH	More effective at higher pH.	Pillinger, Cooper, and Ridge (1994)

*Note:* The use of general terms (high, low, etc.) was an intentional choice. We deliberately made this choice to avoid quantitative thresholds (e.g., lake size, pH) for practices because universal values for all systems and practices do not exist. Instead, the analysis and decision tree are intended to be generalizable based on knowledge of local site conditions and constraints. Please see the SI for some example quantitative thresholds where appropriate for individual techniques.

**TABLE 2 |:** Summary table of physical techniques.

Treatment	Target	Experimental or established?	Factor	Effective conditions	References
Destratification	Nutrients	Established	Clear statement of treatment mechanism	Most effective when tailored to the key mechanism of interest. For example, if internal nutrient load controls are desired, destratification must produce sufficiently high dissolved oxygen concentrations at the sediment water interface.	See references in Supporting Information.
			Mixed or stratified	Effective in stratified water bodies, not mixed water bodies.	See references in Supporting Information.
			Depth	Most effective in waterbodies deeper than 15 m.	Visser et al. (2016); Kibuye, Zamyadi, and Wert (2021b)
Hypolimnetic Oxygenation	Nutrients	Established	Dissolved oxygen concentrations	Nutrient-related benefits of this technique are realized when hypolimnetic oxygenation achieves sediment–water interface dissolved oxygen concentrations > 2mg/L	Preece et al. (2019)
			Source of nutrients	More effective in systems with a predominantly internal nutrient source.	Preece et al. (2019)
			Sediment iron concentrations	The nutrient-related benefits of this technique are realized when sediment Fe:P ratio is 10–15 or higher	Preece et al. (2019)
			Sulfur concentrations	More effective when sulfur concentrations are low.	Preece et al. (2019)
			Nutrient of concern	More effective in treating phosphorus than nitrogen.	Preece et al. (2019)
Dredging	Nutrients	Established	Source of nutrients	More effective in systems with a predominantly internal nutrient source.	Bormans, Maršálek, and Jančula (2016); Riza et al. (2023)
			Treatment area size	More effective in smaller lakes/treatment areas due to cost and level of effort.	Bormans, Maršálek, and Jančula (2016)
Biomass Removal	Cyanobacteria	Experimental	pH	Biomass removal via flocculation is most effective at pH 6.5–9.0	Pan et al. (2006)
			Dissolved organic matter	More effective when dissolved organic matter concentrations are low.	Pan et al. (2006)
			Cyanobacteria cell density	More effective when cyanobacteria cell density is high.	Pan et al. (2006)
			Primary water body purpose	Most appropriate for water bodies used for drinking water and/or recreation and not for those with vulnerable species and/or subsistence resources.	Pan et al. (2006)
Sonication	Cyanobacteria	Experimental	Depth	More effective in deeper water bodies.	Ahn et al. (2003)
			Water clarity	Most effective when water clarity is high.	Vaughan et al. (2023)
			Cyanobacteria community composition	Most effective in removing cyanobacteria taxa with gas vesicles.	Vaughan et al. (2023)
Light manipulation	Cyanobacteria	Experimental	Treatment area size	More effective in smaller lakes.	ITRC (2020)
			Primary water body purpose	Most appropriate for water bodies used for waterbodies with fewer environmental concerns.	Gaskill, Harris, and North (2020)
Flow manipulation	Cyanobacteria	Established	Mixing depth	Vertical mixing more effective in deeper (e.g., > 15 m) water bodies	Visser et al. (2016)
			Treatment area and intensity	Mechanical mixers are most effective when mixing velocities exceed flotation velocities of target taxa and mixers are distributed across the water body	Visser et al. (2016)
			Residence time	Hydraulic flushing is most effective when flushing rate is faster than cell doubling time and circulates flow across the water body	Romo et al. (2013)

**TABLE 3 |:** Summary table of biological techniques.

Treatment	Target	Experimental or established?	Factor	Effective conditions	References
Fish addition or removal	Cyanobacteria	Established	Depth	Less successful in deep water bodies	Scharf et al. (2007)
			Nutrient loads	Biomanipulation is less successful in highly eutrophic lakes due to higher phytoplankton growth rates and because there are more non-edible species, such as colonial or filamentous cyanobacteria.	Kibuye, Zamyadi, and Wert (2021a, 2021b)
					

## Data Availability

Anonymized results from the practitioner survey are available from the lead author upon request.
