# Supplementary material for: A Practitioner-Informed Decision Tree for Selecting Harmful Cyanobacteria Bloom Control and Mitigation Techniques
Source: WIREs Water. Author manuscript; Available in PMC 2025 Mar 7. (PMC11887456; doi:10.1002/wat2.70005)
Supplement: Suppl 1 [file NIHMS2060286-supplement-Suppl_1.pdf]

S1. Overview of treatment methods

Many of the techniques reviewed here require permits or other regulatory approval prior to application and/or implementation. Refer to local regulations for more information.

Biological treatments

Biomanipulation

Biomanipulation is a biological method used to increase pressure on phytoplankton communities by enhancing zooplankton communities through removing/reducing planktivorous fish and/or stocking piscivorous fish. Biomanipulation techniques and effectiveness on reducing cyanobacteria communities have been thoroughly reviewed (e.g., Triest et al. 2016, Kibuye et al. 2021b). The following information summarizes the information contained in these reviews and addresses some recent studies that occurred after these reviews were published.

The most common biomanipulation technique is to remove benthivorous and zooplanktivorous fish to manipulate a waterbody's foodweb (Triest et al. 2016). Removing fish modifies the biological structure of a waterbody by indirectly impacting nutrient cycling and phytoplankton dynamics. Stocking piscivorous fish has some advantages over removing planktivorous fish, but it is usually less successful at achieving improved water quality (reviewed in Drenner and Hambright 1999). More recent work suggests that stocking can be effective if managers stock fish from various trophic levels (Peng et al. 2021, Zhang et al. 2024)

Zhang et al. (2024) found that incorporating a mix of carnivorous, filter-feeding, and detritus-feeding fish led to significant improvements in stability and water quality with Chrysophyta and Bacillariophyta dominating the phytoplankton community. In contrast the control pond in the study with no stocked fish had poorer water quality and the cyanobacteria genera, *Microcystis spp a*nd *Scenedesmus spp* dominated the phytoplankton community. Peng et al. (2021) also had success in utilizing an integrated approach by stocking fish from various trophic levels. Compared to lakes stocked with filter feeding fish or omnivorous fish only, an integrated fish approach was successful at controlling cyanobacteria and overall ecosystem stability. Biomanipulation is less effective in highly eutrophic lakes due to higher algal growth rates and because there are more non-edible species, such as colonial or filamentous cyanobacteria (eppesen et al. 2012, Vašek et al. 2013, Kibuye et al. 2021b). Successful biomanipulation requires external total phosphorus loading to be below ~ 0.6-0.8 g m^2 (Benndorf et al., 2002). If

this threshold is exceeded, the top- down approach of using stocked fish to reduce phytoplankton biomass may be unsuccessful because phytoplankton biomass will likely be controlled through bottom-up processes. In addition to trophic status, other factors that influence performance of biomanipulation include length of the algal growth season, number and type of fish in a waterboy that feed on zooplankton, lake morphology, and environmental conditions (Amorim et al. 2019, Amorim and Moura 2020, Kibuye et al. 2021b).

Biomanipulation in deep waterbodies has often failed or has been less successful than in shallow waterbodies (Scharf et al. 2007). Although there are a few case studies demonstrating success using stocked fish in deeper waterbodies, there is little evidence available to support application of biomanipulation in deep lakes (Vašek et al. 2013). Generally, biomanipulation of deep, stratified waterbodies is considered to be complex and many factors, including controlling external nutrient loading, must be considered before utilizing this technique in these systems. As such, biomanipulation is not commonly used to manage cyanobacteria in deep, stratified waterbodies.

Costs for biomanipulation can vary with the technique applied, size of the water body, and other factors. For example, fish removal can apply different removal techniques (seining, bottom nets, electrofishing, dewatering, varied levels of Rotenone application, etc.). Removal can also occur in phases, via reduction fishing, a more intensive effort that reduces the size of a large population, and/or by maintenance fishing, an approach that involves fishing at a lower rate to maintains a low population. Availability of cost data is limited. Maintenance fishing was conducted at a collection of four shallow (max depth 5m) and small (cumulative surface area of 8.3 km^2) lakes in Sweden. Maintenance fishing was estimated at ~$300,000 for 2022 (Växjö 2021), but costs of initial fish reduction treatments in 1994 to 2000 were unreported. Rotenone treatments for a large scale (50 km^2 reservoir and 260 km of stream) fish removal in Strawberry Valley, Utah cost $3.8M USD in 1990 (Lentsh et al. 2001), though most rotenone projects are much smaller in scale and rotenone is not applied in many areas due to known and substantial ecological risks.

Chemical treatments

Algaecides

Algaecides are chemical solutions that inhibit growth of and/or kill algae and cyanobacteria. Commercially available algaecides include the general categories of 1) herbicides (e.g., Diuron, endothal, atrazine, simazine); 2) oxidants (e.g., hydrogen peroxide products such as PAK-27, GreenClean PRO); and 3) metal-based algaecides (e.g., copper products such as copper sulfate, Captain, K-Tea). More rarely, compounds such as peracetic acid (e.g., VigorOX SP-15, Peraclean, ZeroTol) and chlorine may be

used as algaecides, though there is far less information available regarding the effectiveness and potential consequences of these products. Algaecides are typically applied to the water surface in the presence of cyanobacteria, and frequent reapplication is often necessary to maintain the inhibitory and fatal effect on cyanobacteria. Algaecides affect cyanobacteria through various means, including disrupting electron transport in cyanobacterial cells (copper-based algaecides), inhibiting photosynthesis (hydrogen peroxide and herbicides), and binding to and removing cyanobacteria from the water column via flocculation (clay products) (Buley et al., 2021; Greenfield et al., 2014; Sukenik & Kaplan, 2021). Note that methods involving biomass removal via flocculation are discussed elsewhere in this literature review, and will not be discussed in this sub-section. The relative sensitivities of site-specific cyanobacteria and environmental factors that impact effectiveness of different algaecide formulations including pH, hardness, temperature, particulate and dissolved organic carbon, and cell density can influence the type of algaecide that will be most effective in a given water body (Kinley-Baird et al., 2021).

Herbicides. The most commonly reported herbicides for cyanobacteria control in the literature are Diuron (3-[3, 4-dichlorophenyl]-1,1-dimethylurea) and Endothall (7-oxabicyclo[2,2,1] heptane-2,3-dicarboxylic acid) (Magnusson et al., 2010, Matthijs et al., 2016). However, other herbicides such as cyanazine, diclofop, prometryn, simazine, and simetryn have also been tested for control of cyanobacteria (Teixeira et al., 2020).

Diuron is the only USEPA-approved algaecide approved for use in fish aquaculture and thus, has been widely used to control cyanobacteria blooms while not harming other biota. Diuron acts by inhibiting photosynthesis and has been shown to induce higher toxicity to oxidants relative to many other herbicides (Magnusson et al., 2010). Although this product has been approved for use in aquaculture, degradation products may be a concern during application (Zhou et al., 2013). Furthermore, Matthijs et al. (2016) indicate this compound may persist in sediments over the long-term and is not taxa-selective or specific.

Endothall is also widely used because it is more toxic to cyanobacteria than other phytoplankton; however, it can also exert negative effects on other biota such as zooplankton and fish (Geer et al. 2016; summarized in Matthiljs et al. 2016; Ma et al. 2010). Due to the potential toxicity of this product to fish and zooplankton, endothall is not suggested for widespread usage above 0.3 mg/L. A recent study found that endothall based products were much more effective at controlling *Microcystis aeruginosa* than *M. wesenbergii* suggesting that *M. wesenbergii* may be much more resistant to algaecides and difficult to control than *M. aeruginosa* (Lefler et al., 2022). Endothall based products have also been used in combination with other chemicals, such as hydrogen peroxide based products, to improve overall efficacy. This was exemplified in a laboratory based study that found the endothall-based formulation,

HYDROTHOL® 191 improved the efficacy of the hydrogen peroxide based GreenClean® in decreasing both *M. aeruginosa* and *M. wesenbergii* abundance (Lefler et al., 2022). Finally, Matthiljs et al. (2016) report that long-term application may result in herbicide resistance in targeted cyanobacterial communities.

The use of any herbicide, either alone or in combination with other management techniques, including oxidants and metal-based algaecides, will require a site specific evaluation. Factors such as water chemistry, type of cyanobacteria needing control, size of the system, and sensitivity of other biota must be considered when choosing the appropriate product to apply. Although herbicides are a useful short-term solution for cyanobacteria mitigation there may be negative ecological consequences including killing non-target phytoplankton communities and cyanotoxin release. Utilizing the chemical prior to cyanobacterial blooms may is more effective in controlling blooms and decreases the potential for cell lysis and cyanotoxin release.

Oxidants. The most commonly-used oxidants are hydrogen peroxide-based products. Hydrogen peroxide is considered more ecologically-sound, relative to copper-based algaecides, as it is non-persistent and quickly degrades to oxygen and water (as summarized in Watercourse Engineering 2013, Buley et al. 2021). Numerous laboratory-based studies (e.g., Lusty & Gobler, 2020; Papadimitriou et al., 2022; Spoof et al., 2020; Sukenik & Kaplan, 2021) suggest that hydrogen peroxide applied at concentrations of 1 to 10 mg/L can effectively kill cyanobacteria. Conversely, field experiments demonstrate uncertainty regarding the effectiveness of hydrogen peroxide application. For instance, several studies (e.g., Buley et al., 2021; Watercourse Engineering, 2013) report complete recovery of chlorophyll-*a* concentrations and phytoplankton cell density/biovolume within days of hydrogen peroxide application; Buley et al. (2021) noted that granular forms of hydrogen peroxide were more effective in maintaining lower phytoplankton biovolume post-application, relative to liquid forms. Yang et al. (2018) reported that a dose of 6.7 mg/L effectively eliminated several cyanobacteria genera for the duration of a 7-day field experiment. Barrington et al. (2012) concluded that hydrogen peroxide application decreased cyanobacteria biomass over a three-week period in a waste stabilization pond.

There is uncertainty around the effect of hydrogen peroxide on cyanotoxin concentrations, and how different cyanobacterial taxa respond to treatment. For instance, Greenfield et al. (2014) and Spoof (2020) concluded that hydrogen peroxide at best does not have an effect on cyanotoxin concentrations, and at worst, may increase concentrations of dissolved cyanotoxins as a result of cell lysis. Conversely, Watercourse Engineering (2013) reported decreases in microcystin concentrations of 11 to 80 percent following hydrogen peroxide application in a reservoir. In terms of taxa responses, *Planktothrix* showed a high degree of sensitivity, while *Microcystis*, *Pseudanabaena*, and *Raphidiopsis* were moderately sensitive, and *Cylindrospermopsis* was least sensitive

(Lusty & Gobler, 2020; Sandrini et al. 2020; Weenink et al. 2021, as summarized in Kibuye et al. 2021a;). Toxic strains of *Microcystis* appear to be less sensitive to hydrogen peroxide than non-toxic strains (as summarized in Kibuye et al., 2021a). Finally, additional research is necessary to determine if hydrogen peroxide is an effective treatment for benthic cyanobacteria (Kibuye et al. 2021a).

Costs for hydrogen peroxide application vary by dose, but tend to range from \$0.20 to \$0.36 per m^3, including labor (2013 US dollars; Watercourse Engineering 2013). It is important to note that frequent reapplication (e.g., every couple days to once per month; Barrington et al., 2013; Watercourse Engineering, 2013) is likely necessary to control cyanobacteria biomass. Targeting cyanobacteria bloom “hot spots” within a water body may offer an effective solution to cyanobacteria issues at a more reasonable cost.

Additional considerations for hydrogen peroxide application include unintended consequences and factors limiting the effectiveness of this treatment. It is possible that algaecides, including hydrogen peroxide, may result in substantial decreases in dissolved oxygen and increases in unionized ammonia concentrations due to decomposing cyanobacteria biomass following treatment (as summarized in Buley et al. 2021). Furthermore, Watercourse Engineering (2013) observed increases in orthophosphate concentrations immediately following hydrogen peroxide application, likely as a result of cyanobacteria decomposition, suggesting this technique could increase the amount of phosphorus available to cyanobacteria and other phytoplankton species. Indeed, Buley et al. (2021) reported that liquid hydrogen peroxide application resulted in a phytoplankton community shift towards cyanobacterial dominance at the conclusion of a 35-day field experiment. Additionally, there is uncertainty regarding potential effects of hydrogen peroxide on non-target organisms. Yang et al. (2018) reported that applications of hydrogen peroxide at 6 mg/L resulted in minimally negative effects to zooplankton. In contrast, several studies (Buley et al. 2021; Reichwaldt et al. 2012; Spoof et al. 2020) indicated this technique resulted in depressed zooplankton and eukaryotic algae (non-cyanobacteria phytoplankton) densities at doses well within the range typically recommended for treatment. Relative to treatment effectiveness, light, wind, and algal composition and density (e.g., greater chlorophyta or cyanobacteria densities) can affect performance (Barrington et al., 2013; Drábková et al., 2007; Piel et al., 2019; Weenink et al., 2021). Furthermore, colored dissolved organic matter (typically associated with wetlands and other systems producing tannins and similar organic compounds) is known to speed degradation of hydrogen peroxide, particularly under high light conditions (Paerl & Otten, 2013a). Conversely, there is some suggestion that higher solar irradiance may accelerate the formation of hydrogen peroxide byproducts responsible for the algaecidal effects of this treatment method (as summarized in Kibuye et al., 2021a).

In conclusion, hydrogen peroxide application is a very promising option for short-term and immediate treatment of cyanobacteria blooms in freshwater. However, long-term effectiveness and potential impact on biodiversity, including that of zooplankton and non-target phytoplankton species, need further study and consideration.

Metal-based algaecides. Copper products reduce phytoplankton biomass immediately after application (Albay 2003, Watercourse Engineering 2013, Greenfield et al. 2014, Buley et al. 2021), though chlorophyll-a concentrations and cyanobacteria biovolume returned to pre-treatment concentrations within days to weeks, particularly in field experiments (Buley et al., 2021; Hanson & Stefan, 1984). Copper-based algaecides do not appear to have an effect on cyanotoxin concentrations, but high doses, particularly necessary in scenarios with high cyanobacteria cell density, inducing cell damage may result in increased dissolved toxin concentrations (as summarized in Kibuye et al. 2021a). Given the concerns expressed below and the general abandonment of copper-based algaecides for use in cyanobacterial bloom control, we did not attempt to estimate cost or labor associated with application of these products.

Additional considerations for copper-based algaecides include unintended effects, and factors effecting performance. It is well established that copper-based algaecides have substantial negative effects on non-target organisms (e.g., zooplankton, fish, and/or algae) immediately after application, and likely over the long-term. For instance, Williams et al. (2015) report a 93 percent reduction in zooplankton biomass and density immediately after copper sulfate application in a drinking water reservoir. Similarly, Hanson & Stefan, (1984) indicate copper sulfate application had dire long-term consequences for zooplankton and fish populations in five Minnesotan lakes. Moore & Winner (1989) further conclude that macroinvertebrates (e.g.,chironomids) are susceptible to copper toxicity. Additionally, it is possible that algaecides, including copper products, may result in substantial decreases in dissolved oxygen concentrations, increases in unionized ammonia concentrations, and changes in nutrient fractions (e.g., from total to dissolved fractions) due to decomposing cyanobacteria biomass following treatment. Relative to factors affecting copper algaecide performance, pH between 5 and 7, decreasing suspended solids and organic matter concentrations, increasing alkalinity; lower cyanobacteria cell densities; and water temperatures above 10°C appear to increase copper toxicity, especially for *Microcystis aeruginosa* (as summarized in Kibuye et al., 2021a; Raman & Cook, 1988; Stroom & Kardinaal, 2016; Zeng et al., 2010). High light intensity appears to increase the effectiveness of copper algaecides (as summarized in Greenfield et al., 2014; Watercourse Engineering, 2013).

Generally, copper-based algaecides are no longer recommended for remediation of cyanobacteria blooms. However, there may be some utility in specific situations in which there are minimal concerns regarding effects to non-target species and the

potential for sediment copper accumulations (e.g., in water bodies managed solely for recreation or drinking water).

Algal scrubbers

Algal scrubbers are engineered systems resembling continuous flow hatchery raceways inoculated with benthic algae and periphyton, and used for nutrient removal. Commercially, these systems are known as Algal Turf Scrubbers, or ATS^{TM}. The primary mechanism driving nutrient removal in algal scrubber systems involves assimilation of P and N into attached algal and periphyton biomass, which is then routinely removed from the raceway (as summarized in Adey et al., 2013). Additionally, the complex surface associated with attached algae and periphyton facilitates deposition of particulate nutrients in the raceways. Finally, precipitation of calcium phosphate also effectively removes P from inflowing water when pH is elevated (i.e., around 8; Ferguson et al., 1973) as a result of photosynthesis. Frequent (e.g., weekly; Adey et al., 2011; Dinkins et al., 2009; Mulbry et al., 2010) removal of algal and periphyton biomass, primarily via vacuum, is necessary to maintain nutrient removal and algal growth rates. Increasing the topographic complexity of raceway substrate substantially increases algal biomass in the scrubber system (Adey et al., 2011).

In two small (24 m^2 total surface area) algal scrubbers deployed in the Chesapeake Bay area, Adey et al. (2013) observed particulate P, TDP, and TP mean percent removal rates of 63, 43, and 51 percent, respectively. N removal rates were much lower at 50, 12, and 25 percent for particulate N, TDN, and TN, respectively (Adey et al., 2013). Mulbry et al. (2010) reported maximum annual average removal rates of 6 g TP/m^2/year and 31 g TN/m^2/year in small (1 m^2 each) algal scrubbers in the Chesapeake Bay watershed; the authors noted that TP and TN removal rates were on average 11 and 6 times lower, respectively, during the winter, relative to those observed during the growing season. Dinkins et al. (2009) estimated algal scrubbers deployed to treat stormwater in Florida removed 92 g TP/m^2/year (23 percent of inflow TP) and 727 g TN/m^2/year. Algal scrubbers in central California and southern Florida removed an average of 255 and 37-51 g TP/m^2/year, respectively (Adey et al., 2011). (Adey et al. (2011) also reported N removal rates of 80 to 100 percent when algal scrubbers were used to treat concentrated animal waste. It appears that removal rate increases with increasing inflow nutrient concentrations and load (Adey et al., 2011). Finally, Adey et al., (2011) reported that algal scrubber systems deployed in southern Florida have two orders of magnitude greater P removal rates than nearby managed wetlands.

Labor associated with biomass removal in algal scrubbers is likely substantial. In relatively small algal scrubber systems (24 m^2 total surface area), Adey et al. (2013) observed that biomass removal required approximately 7.5 minutes per square meter of raceway, with the frequency of removal dependent on growth rates and project objectives. Generally, algal scrubber systems are considered at least as cost effective as

techniques such as engineered water quality treatment wetlands, when costs are averaged over 50 years of operation (Adey et al. 2011); this assessment did not account for capital necessary to acquire land and construct algal scrubber systems. Regardless, the biomass resulting from algal removal, and the frequency of removal efforts, is expected to increase with increasing inflow nutrient concentration and load (Adey et al. 2011).

Additional considerations include growing season length, winter maintenance concerns, and potential for revenue-generating end uses. Given the primary nutrient removal mechanism associated with algal scrubbers, this technique is most effective during the growing season, with the potential for limited nutrient removal during the winter. In areas with more severe winters, algae and periphyton are likely to slough from raceways, simplifying the raceway substrate and flow patterns, and reducing the potential for particulate P deposition. Similarly, with a reduction in photosynthesis in the winter (and associated decrease in pH), P precipitation is likely negligible outside of the growing season in temperate climates. Indeed, algal scrubbers have been most commonly implemented for the purpose of nutrient removal in Mediterranean (e.g., studies summarized in Adey et al. 2011) and sub-tropical (e.g., Adey et al., 2011; Dinkins et al., 2009) climates with lengthy growing seasons; average annual nutrient removal potential is expected to be substantially lower in temperate regions. Additionally, algal scrubbers deployed in regions with cold winters may require full removal or substantial maintenance due to snow and ice accumulation that could damage raceways and algal substrate. Finally, it has been suggested that removed algal biomass can be used as livestock feed, soil amendments, and health supplements, and for the production of biofuels (as summarized in Adey et al. 2013; Dinkins et al. 2009). It is unclear what demand and market exist for these end uses.

Barley straw

This technique involves deploying barley straw, typically as bales or bagged in fabric netting, in water bodies to inhibit growth of nuisance algae and cyanobacteria. Barley straw is most often deployed in relatively small water bodies, such as ponds and canals (Ferrier et al. 2005). The mechanisms underlying the inhibitory effect of barley straw are not fully understood, but generally involve the release of dissolved and oxidized compounds during and after aerobic decomposition of barley straw (as summarized in Boylan & Morris, 2003; Pillinger et al. 1994). Recommended barley straw doses appear to range widely, but are generally between 11 - 44 g/m^2e (Ferrier et al. 2005; Islami & Filizdeh, 2011). The inhibitory effect is only maintained if barley straw is replaced as it decomposes; the frequency of reapplication is dependent on temperature and dissolved oxygen concentrations (i.e., the variables controlling decomposition rates), and could range from weekly to monthly. Additionally, inhibitory compounds are only present under aerobic and basic conditions (as summarized in Pillinger et al. 1994); this method

is unlikely to produce desired inhibitory effects in hypoxic and anoxic systems (Boylan & Morris, 2003). Several studies (e.g., Boylan & Morris, 2003; Pillinger et al. 1994) recommend placing barley straw near inlets or in other areas with elevated dissolved oxygen concentrations.

The majority of studies examining the effects of barley straw on algae and cyanobacteria do not include replication and/or a suitable before-after-control-impact study design (as described in Ferrier et al. 2005). As such, it can be difficult to attribute study findings to the effect of barley straw alone. Regardless, there appears to be wide agreement in the literature that compounds produced during and after barley straw decomposition have a strong and statistically significant inhibitory effect on the growth of cyanobacterium *Microcystis aeruginosa* (Ferrier et al. 2005; Islami & Filizdeh, 2011; Pillinger et al. 1994). Additionally, Caffrey & Monahan (1999), reported a statistically significant reduction in biomass of unspecified filamentous algae. In other cases, barley straw stimulated, did not affect, or had varying (stimulatory in some studies, inhibitory in others) effects on algae and cyanobacteria growth (Pillinger et al. 1994, Caffrey and Monahan 1999, Boylan and Morris 2003, Ferrier et al. 2005 and studies summarized therein, Islami and Filizadeh 2012). The majority of studies reporting measurable and statistically significant inhibitory effects are lab-based, with relatively few demonstrating success in field applications (Ferrier et al. 2005).

Given that the majority of barley straw applications involve relatively small waterbodies such as ponds and canals, labor and costs associated with this technique are likely minimal, especially compared with other methods described in this literature review. However, barley straw application (and reapplication/replacement) in larger systems and at recommended doses reported above would like require a substantial amount of labor.

Additional considerations include the potential for increased orthophosphate and cyanotoxin concentrations resulting from barley straw application, and the need for guidelines related to effective straw dosage levels (as summarized in Lurling et al. 2016 and Kibuye et al. 2021b). Generally, this technique requires additional rigorous assessment to address uncertainties and the mixed results reported in the literature.

Floating wetlands

Floating wetlands have been implemented worldwide with the objective of reducing heavy metal, toxin, chlorophyll-*a*, suspended sediment, and nutrient concentrations in water bodies (Masters, 2012; Zhang et al. 2014). Floating wetlands typically consist of a buoyant floating platform planted with emergent wetland plant species such that plant roots are suspended within the water column below the platform. Platform materials and plant species vary widely depending on project objective, geographical location, and budget (Masters, 2012; Zhang et al. 2014). Key nutrient sequestration mechanisms associated with floating wetlands include dissolved nutrient assimilation in plant and

periphyton biomass (Sample et al. 2013; S. A. White & Cousins, 2013; Zhang et al. 2014), and deposition of particulate nutrients (Masters, 2012; Sample et al. 2013; Zhang et al. 2014) as hydraulic residence time increases in and around floating wetlands. Most floating wetlands require removal of above-water biomass at the end of the growing season to prevent release of nutrients upon senescence and subsequent decomposition (Garcia Chance et al. 2019; Sample et al. 2013; S. White et al. 2009). Additionally, it may be necessary to remove (e.g., dredge) particulate matter deposited in the vicinity of floating wetlands to prevent mineralization and eventual release of orthophosphate (orthoP) and dissolved nitrogen (N) forms, though it is unclear what magnitude of released nutrients would be assimilated by floating wetlands.

Lynch et al. (2015) and Spangler et al. (2019) reported mean gross floating wetland nutrient removal rates of 2.8 g TP/m^2/year and 9.5 g TN/m^2/year, and 2.7 g TP/m^2/year, respectively, over a nine-week growing season. Removal rates were dependent on inflow nutrient concentrations, plant species associated with the floating wetlands, and brand of commercial floating wetland tested (Lynch et al. 2015; Spangler et al. 2019). In mesocosms containing floating wetlands and aeration systems, and with elevated nutrient concentrations (i.e., 15 mg orthoP/L, 250 mg nitrate/L), Stewart (2007) and Stewart et al. (2008) noted maximum gross removal rates of 1,680 g orthoP/m^2/year, and 720 g orthoP/m^2/year, 38,690 g TN/m^2/year, and 996 g ammonia/m^2/year, respectively, over two 22-hour study periods. However, tanks containing floating wetlands, but no aeration, had orthoP concentrations similar to or slightly higher than control tanks (F. Stewart, 2007; F. Stewart et al. 2008). Generally, it appears that nutrient removal rates associated with floating wetlands may be substantially greater in the few hours and days immediately following implementation (F. Stewart, 2007; F. Stewart et al. 2008), removal rates are positively correlated with nutrient concentration in the surrounding water (Garcia Chance et al. 2019; Pavlineri et al. 2017; S. White et al. 2009), aeration is likely necessary to achieve positive P removal rates (F. Stewart, 2007; F. Stewart et al. 2008), and removal efficiencies may not be notably different between treatment and control ponds/tanks (Lynch et al. 2015; Stewart et al. 2008). Additional study is necessary to adequately assess P removal rates associated with floating wetlands, but this technique appears relatively effective for N treatment.

Cost estimates for commercially-produced floating wetlands (including installation, but not always including plants) typically range from \$270 to \$380 per m^2 (Lyon et al. 2009; Sample et al. 2013). "Homemade" floating wetlands typically cost \$32 per m^2 for purchase of supplies and subsequent installation (Lyon et al. 2009), not accounting for inflation and other cost increases since the 2010s. It is also important to consider the potentially substantial level of effort necessary to remove biomass from and maintain floating wetland platforms.

Additional considerations include growing season length, winter maintenance concerns, implications for underlying water chemistry, and creation of bird habitat. Given the primary nutrient removal mechanism associated with floating wetlands, this technique is most effective during the growing season, with the potential for limited nutrient removal during the winter. In areas with more severe winters, wetland vegetation and periphyton are likely to senesce and slough from floating platforms, simplifying flow patterns, and reducing the potential for particulate nutrient deposition. Indeed, floating wetlands have been most commonly implemented to treat nutrients in sub-tropical climates with lengthy growing seasons (e.g., those described in Pavlineri et al. 2017); average annual nutrient removal potential is expected to be substantially lower in temperate regions. Additionally, floating wetlands deployed in regions with cold winters may require full removal or substantial maintenance due to snow and ice accumulation that could damage platforms and plant roots. Another important consider is the potential for floating wetlands to decrease dissolved oxygen concentrations in the water column below and adjacent to the platforms (Lynch et al., 2015; F. M. Stewart et al., 2008); this is likely due to an increase in organic matter deposition and decomposition below floating wetlands coupled with a decrease in photosynthesis in the water column associated with platform shading (as summarized in Skinner, 2020). Finally, floating wetlands may increase habitat for birds such as pelicans, cormorants, and waterfowl (as summarized in Skinner, 2020).

Phosphorus immobilization (alum, calcium, iron, lanthanum bentonite)

Nutrient inactivants are used primarily to control the internal cycling of P within a waterbody, but can also be used to strip P from the water column or inflows to a water body. Nutrient inactivants are a popular lake management intervention because they can be utilized in waterbodies of varying sizes and eutrophication states (Copetti et al., 2016; Lürling et al., 2020; Mucci et al., 2020). Commonly-used nutrient inactivants include hydrated aluminum sulfate (alum), bentonite clay infused with lanthanum (commercially available as Phoslock™), modified Zeolite (Z2G1), ferric salts or liquid $Fe_2(SO_4)_3$, and calcite or lime. An increasing number of novel materials are also being proposed for use in lakes and reservoirs (Hickey & Gibbs, 2009). Nutrient inactivants sequester P from the water column through precipitation, forming a heavier than water particulate known as a floc. As the floc settles it, removes P and suspended particles[1] from the water column and carries them to the bottom of the waterbody. The floc forms a layer on top of the sediment that acts as a barrier by adsorbing P that is released from the sediments during periods of hypoxia or anoxia (Copetti et al., 2016; Gibbs et al., 2011; Ross et al., 2008). With the exception of treatments primarily composed of iron, this floc barrier is insoluble in water, even under anoxic conditions.

[1] Flocculants used for nutrient control may also result in removal of cyanobacteria and algal cells from the water column. Refer to the biomass removal section for additional detail.

The properties of different nutrient inactivants and their effectiveness under a range of physicochemical parameters are reviewed in various papers (Copetti et al., 2016; Douglas et al., 2016; Hickey & Gibbs, 2009; Kibuye et al., 2021a; Lürling et al., 2020; Mucci et al., 2020; Zamparas & Zacharias, 2014). The efficacy of nutrient inactivants is a function of the morphological and physicochemical characteristics a waterbody. Key factors that influence dosing and subsequent effectiveness of nutrient inactivants in a waterbody include depth, residence time/hydraulic flushing, the period and depth of stratification, wind fetch, water quality (e.g., alkalinity, pH, suspended solids, dissolved constituent content), and sediment characteristics (Hickey & Gibbs, 2009). A combination of these factors and treatment characteristics (e.g., area treated, application technique, formulation) influence the outcome of nutrient inactivant application. As an example, the formation of alum floc is dependent on high alkalinity, either natural or supplied via a buffer added during dosing (Gibbs et al., 2011).

Nutrient inactivants that settle as a capping layer on the sediment can remain effective for 5 years and up to 20 years in some lakes (Welch & Cooke, 1999) before repeat treatment is needed. Alum treatment has been shown to have a mean treatment longevity of 21 years in deeper, stratified lakes and a mean of 5.7 years in shallow, polymictic lakes (Huser et al., 2016). Lanthanum modified bentonite has also shown long-term effectiveness in lake restoration (i.e., up to 10+ years; Copetti et al. 2016).

Alum and lanthanum-modified bentonite have been used and studied for nutrient control more than calcium and iron. However, both iron and calcium have been effective at controlling P, at least for short period of time. Calcium treatments generally provide shorter-term improvements than alum and lanthanum modified bentonite as precipitating salts redistribute as they settle onto the sediment floor (Leoni et al. 2008, Kibuye et al. 2021a). When calcium treatments are combined with alum, the treatment longevity appears to improve (Mehner et al. 2008). Even fewer studies have evaluated the longevity of iron treatment. Nevertheless, iron treatment has also been found to control P accumulation in the hypolimnion for months to years depending on the amount of external P loading that continues to occur during treatment (Quaak et al., 1993, Kleeberg et al., 2013, Gołdyn et al. 2014). Forecasting the length of time for an ecological response to all types of nutrient inactivant applications remains challenging.

The greatest issue associated with nutrient inactivants is that incorrect dosing can cause short-term undesirable environmental effects on water quality and biota (Cooke et al., 2005). Other downsides to the tool include the need, in some waterbodies, to reapply nutrient inactivants several times a year.

Nutrient inactivants are most effective when external nutrient load is controlled. Even in waterbodies that require less frequent dosing, over time the floc layer becomes buried by detritus. As the detritus decomposes, it can refuel nutrient release from the sediments (Cooke et al., 2005; Welch & Cooke, 1999). The mineralization rate of organic P in the sediment often determines the frequency and concentration of nutrient

inactivant needed to suppress P diffusion out of the sediments. Some laboratory studies have also shown that, once nutrient inactivants settle onto the sediment, benthic microbial processes of nitrification and denitrification can be disturbed (Gibbs et al., 2011).

Finally, field-scale studies using nutrient inactivants report very limited, if any, negative ecosystem effects, but many of these studies lack sufficient monitoring to adequately assess post-treatment ecological impacts (Kibuye et al. 2021a). A number of lab and mesocosm studies report negative effects ranging from declines in non-target algal communities, aquatic vegetation, and fish to bioaccumulation in aquatic organisms that potentially affects higher trophic levels (Kibuye et al. 2021a). There is also some indication that nutrient inactivant overdosing may result in damage to the cells of toxin-producing cyanobacteria, resulting in increased concentrations of dissolved cyanotoxins following treatment (as summarized in Kibuye et al., 2021a).

There are currently no criteria available to select the most appropriate nutrient inactivant for a particular waterbody. Thus, it can be difficult to make recommendations to managers on the best option for their lake. However, cost may be the driving factor in choosing which nutrient inactivant to utilize. Costs fluctuate widely depending on the nutrient inactivant of choice, the size of the waterbody, and management goals. For example, Phoslock™ is of the order of thousands of dollars per ton, which is approximately one order of magnitude higher than the cost of alum (Copetti et al., 2016). Yet, especially in smaller water bodies, application of Phoslock™ or other lanthanum-based materials may be very cost effective. In Australia, studies showed an immediate reduction (i.e., 95 to 99 percent) in phosphorus in small, cyanobacteria-prone waterbodies for approximately $150,000 per application (Douglas et al., 2016). While there are many remaining questions on determining the type, amount, and frequency of nutrient inactivant applications, these materials continue to serve as the best option for addressing internal nutrient cycling (Douglas et al., 2016).

Physical treatments

Biomass removal

Algal/cyanobacterial biomass removal is a remediation technique involving physical or chemical biomass removal from a body of water. Chemical techniques largely involve the use of algaecides or the chemicals utilized for phosphorus immobilization, which are discussed elsewhere in this review. Physical methods include filtration (through a net or similar), suction (e.g., using a vacuum system), and flocculation (Stroom & Kardinaal, 2016). Flocculant agents result in clumping and settling of cyanobacteria cells (Stroom & Kardinaal, 2016); this method is by far the most assessed in the literature and will be the primary focus of this sub-section.

Compounds that result in direct flocculation of, and decreases in, cyanobacteria biomass include local soils amended with chitosan, iron chloride, ferrous chloride, iron sulfate, and alum (summarized in Stroom & Kardinaal, 2016). Many of these compounds also bind and sequester phosphorus (i.e., see phosphorus immobilization section above), indirectly affecting cyanobacteria biomass over longer time scales. Thus, frequent reapplication may prevent cyanobacteria blooms from occurring (Stroom & Kardinaal, 2016).

Flocculants including sepiolite, talc, ferric oxide, and kalinite clays dosed to 0.7 g/L removed 90 percent of *Microcystis aeruginosa* biomass 8 hours after application in a lab-based assessment (Pan et al., 2006a); similar removal rates were observed for sepiolite at a dose of 0.2 g/L (Pan et al., 2006a). A subsequent study amended numerous clays and local soils/sediments with chitosan (a synthetic compound derived from shellfish chitin), resulting in greater than 90 percent biomass removal at a dose of 0.011 g/L (containing 10 percent chitosan) (Zou et al., 2006). Finally, Pan et al. (2006b) reported that the removal of *Microcystis aeruginosa* biomass by chitosan-amended sepiolite and kaolinite was maximized at pH 6.5 to 9.0, with decreasing salinity and humic acid concentrations, at cell concentrations above 5×10^3 cells/mL, and during early bloom senescence stages (when cyanobacteria cell concentrations tend to be highest). A simultaneous *in situ* experiment in Lake Taihu, China with chitosan-amended local soils/sediments (dose = 0.025 g/L, equivalent to 50 g/m^2; containing 10 percent chitosan) resulted in a 99 percent decrease in chlorophyll-*a*, with no observed increase in chlorophyll-*a* for the duration of the month-long post-treatment monitoring period (Pan et al., 2006b).

Filtration and suction appear to be far less effective in removing cyanobacteria biomass, and especially in maintaining low biomass post-treatment. Indeed, removal of 30 to 60 percent of *Microcystis* sp. biomass from mesocosms via filtration resulted in increased growth and biomass recovery within 6 days of removal (Fan et al. 2019). Conversely, 90 percent biomass removal resulted in statistically significant reductions in *Microcystis* sp. biomass, relative to the control, for 15 days post-treatment (Fan et al. 2019). Continuous biomass removal led to increased cyanobacteria diversity, resulting in proliferation of species that were more difficult to remove via filtration (Fan et al. 2019). These findings indicate that biomass removal via filtration is only likely to have a lasting effect if efforts are efficient and continuous. There is a distinct lack of quantitative and peer-reviewed assessments for suction methods.

Additional considerations for biomass removal to control cyanobacteria and harmful algal blooms include uncertainty around cost and frequency of application/implementation, decomposition of flocculated biomass, unintended affects to zooplankton and desirable algae, and concerns around disposal of biomass obtained through filtration (due to potentially high cyanotoxin concentrations in removed biomass). Relative to decomposition, it appears that flocculation of cyanobacteria

biomass and subsequent decomposition may result in elevated dissolved nutrient (Pan et al., 2006b) and extracellular cyanotoxin concentrations (Mucci et al., 2020), and low dissolved oxygen concentrations (Liu et al., 2022; Pan et al., 2006a). While it is unclear if these effects would be greater in magnitude than what is observed over the typical course of a cyanobacteria bloom, concentrating senescence of the bloom into a relatively short time period via flocculation may change the magnitude and timing of these water quality effects. In addition to concerns regarding the effect to water quality, flocculation may also decrease zooplankton and non-cyanobacteria phytoplankton biomass (Peng et al. 2019), eliciting potentially negative and unintended consequences for non-target organisms. Finally, for methods that involve removing biomass entirely from water bodies (e.g., filtration and suction methods), there are concerns around disposal and end-uses. In particular, cyanotoxins are notoriously persistent, and cyanobacteria biomass therefore needs to be handled with care (Stroom & Kardinaal, 2016). Furthermore, there tend to be limited end-uses for removed biomass (Stroom & Kardinaal, 2016) due to variable quality, and presence of non-target species and cyanotoxins (especially concerning for pharmaceutical products and biofuel).

Destratification

Destratification can be effective for suppressing HABs in deep, stratified water bodies. Artificial mixing systems promote destratification by moving water with pumps, or by injecting air via bubble plumes or curtains (referred to as diffusers) (Jungo et al. 2001). These systems are typically operated from the onset of thermal stratification in the spring through turnover in the fall. The purpose of destratification in this context is to decrease cyanobacteria growth and dominance by a) eliminating cyanobacteria competitive advantage of buoyancy regulation and vertical migration, b) making conditions more favorable for other algae (via reduced sedimentation rates for non-buoyant algae; (Visser et al. 2016), which are much more successful in low light than cyanobacteria; and c) bringing cold hypolimnetic water to the surface, reducing total productivity for all phytoplankton, though with a greater impact to temperature-sensitive BGA (Kibuye et al. 2021b). In addition, limited research has demonstrated that increasing dissolved oxygen concentrations in the water via mixing may also improve conditions for the zooplankton that graze on BGA (Becker et al., 2006). Furthermore, destratification may decrease water column nutrient concentrations if it promotes higher dissolved oxygen concentrations and redox in the hypolimnion (Kibuye et al. 2021b).

Destratification systems can be implemented as curtain or plume diffusers that are suspended or anchored at some distance from the bottom (e.g., 1 m, Seelos et al., 2021) of deep lakes and reservoirs. The deployment depth, or distance of the diffuser from the bottom, is an important design component (Chen et al., 2018), since the diffusers also affect the profundal water chemistry and microbial processes in complex

ways (Seelos et al., 2021). This practice was reviewed by (Kibuye et al., 2021b), who reported on multiple successful examples of aeration systems when mixing is strong and deep, and highlighted how the effectiveness of mixing varies with a taxa's floatation velocities and light limitation varies among cyanobacteria species. While general design guidance for destratification exists (e.g., generally effective in lakes > ~15 m deep, maximizing deployment depths of bubble diffusers for destratification, etc.), design of destratification systems requires detailed data and modeling of the hydrodynamics. Additional considerations for artificial mixing systems include unintended ecosystem consequences. Destratification may result in increased internal nutrient load if biomass sedimentation and associated sediment oxygen demand from decomposition exceeds improvements in dissolved oxygen via destratification, or if mixing actively resuspends nutrients from the sediment to water column (as summarized in Kibuye et al. 2021b). Artificial mixing may also result in increased availability of dissolved inorganic carbon (Verspagen et al. 2014), which can stimulate phytoplankton growth. Finally, destratification typically results in warmer water temperatures in deeper water strata, which may have a negative effect on coldwater fish (as summarized in Kibuye et al. 2021b).

Costs of destratification systems will vary substantially with the size of the lake, which drives the system design and the pumping rates. Both capital costs (e.g. pump(s), on-shore compressor, conduits, diffuser, etc.) and ongoing operational costs (e.g. power demands, maintenance) are expected can be large for anything but small lakes. At Sweetwater Lake, an approximately 35 million m^3 drinking water supply reservoir in CA, was projected to cost $1.2 – 1.5 million in 2023 (Block & Adam, 2022).

Dredging

Dredging is a technique that aims to decrease internal nutrient cycle by excavating nutrient-rich sediments and then relocating the sediments to a disposal site, preferably outside of the immediate watershed to prevent re-entrainment of nutrients in the target water body. Dredging can also be used to remove cyanobacteria resting cells and organic carbon. Due to high costs and the ecological impacts of sediment disturbance (e.g., reductions in benthic macroinvertebrates and aquatic vegetation; as summarized in Kibuye et al. 2021), dredging is viewed as a controversial mitigation technique (Jing et al., 2019; Kiani et al., 2020; Paerl & Barnard, 2020). Reports on the effectiveness of dredging are varied with some claiming high success and others finding little to no improvement in water quality. Generally, successful cases involve relatively small lakes for which external nutrient loading has been controlled (Bormans et al., 2016). (Kibuye et al., 2021a) and Riza et al. (2023) provide a comprehensive review of successful and unsuccessful dredging projects. Researchers generally agree that dredging can be an effective method to prevent or mitigate cyanobacteria blooms in systems meeting specific criteria, but that it critical to also control external nutrient loading for true

success (Riza et al., 2023). Further costs and ecological impacts must be weighed when choosing this option.

Flow manipulation

Flow manipulation herein refers to practices associated with modifying hydrodynamics that does not aim to destratify the system (described separately below). The practices can include less aggressive vertical mixing, horizontal flushing, and potentially epilimnetic mixing.

Visser et al. (2016) reported a comprehensive summary of vertical mixing as a method to control cyanobacterial blooms. Numerous studies indicate that mixing can be effective in shifting phytoplankton community assemblage from dominance by cyanobacteria to green algae and diatoms if the mixing is strong and deep enough to entrain the BGA in the turbulent flow and create light limiting conditions (Visser et al. 2016; Kibuye et al. 2021b). However, artificial mixing does not appear successful in supressing cyanobacteria growth and/or shifting phytoplankton assemblages in systems where vertical mixing is inadequate, the lake is shallow, the distribution of mixers across the lake is insufficient, or in systems with uncontrolled external nutrient loading (as summarized in Kibuye et al. 2021b). (Visser et al. 2016) indicates that well-mixed conditions suppressing BGA growth can be generated when the ratio of mixing depth to euphotic depth greater is than 3, and when the lake depth is greater than ~15 m. In some cases, continuous or intermittent mixing in lakes with depths <15 m can enhance algal growth since it is not likely to create light limitation and can circulate P and N from the bottom sediments. This phenomenon may explain why epilimnetic mixing has had such a limited record of success. Vertical mixing must also be aggressive enough to match or exceed the floatation velocities in target cyanobacteria taxa (Kibuye et al. 2021b). For instance, colony-forming taxa (e.g., *Microcystis*) have much higher average floatation velocities than filamentous cyanobacteria (e.g., *Anabaena*), which has implications for design specifications (as summarized in Kibuye et al. 2021b). Finally, intermittent, rather than continuous, mixing is considered in some systems where energy savings is important, though intermittent operations has shown mixed success and can actually promote cyanobacteria productivity, depending on the target cyanobacteria species, the timing of mixing, and the limnetic conditions (see reviews in Kibuye et al. 2021b and Visser et al. 2016).

Hydraulic flushing is another flow modification strategy for suppressing HCBs. The primary goal is to reduce the residence time of a water body. Residence time refers to the length of time that water (or sediment, dissolved substances, and algae) spends in

a lake. The distribution of residence times across lakes globally ranges from hours to decades, with a median residence time of 456 days (Messager et al. 2016). However, residence time varies substantially, where large lakes with little discharge have exceptionally long residence times and smaller lakes connected to rivers may regularly experience flushing and hence short residence time. Residence time is important because cyanobacterial growth is favored under conditions associated with long residence times. This is a result of relatively (compared to eukaryotes) low growth rates of cyanobacteria (Ralston et al., 2015; Romo et al., 2013). Reducing residence times can suppress cyanobacteria growth via flushing of algal biomass, delaying stratification, suppressing internal loading, and disrupting vertically migrating cyanobacteria (Olsson et al., 2022). From a design perspective, hydraulic flushing can involve diverting flow from an adjacent water source and is most effective when the flushing rate is faster than the cell doubling time and circulation extends across the lake. Flushing requires an alternate water source that may not exist at most sites. This practice has been recommended at the Ross Island lagoon on the Lower Willamette River, Oregon, where a former gravel operation and land modifications resulted in a 37 m-deep pool in the middle of the river that hosts increasingly severe toxic cyanobacterial blooms. Costs for these projects will vary substantially across sites, but generally involve channel excavation, channel bed and bank materials, and water control infrastructure.

<u>Hypolimnetic oxygenation</u>

Hypolimnetic oxygenation involves the injection of pure oxygenation into water, typically while maintaining thermal stratification (as summarized in Kibuye et al. 2021a). This technique is employed to increase the dissolved oxygen concentration in deep waters and is primarily deployed in deep, thermally stratified lakes. Although there are a number of potential reasons to implement hypolimnetic oxygenation (e.g., to improve color, taste, and odor of drinking water; to improve coldwater fish habitat in deep lake waters), the purpose most relevant to this literature review is the use of the technique to minimize chemical reactions within the sediment and sediment water interface that lead to internal nutrient loading. Specifically, increasing dissolved oxygen concentrations at the sediment water interface increases oxidation-reduction potential (i.e., "redox" or ORP), which in turn decreases the likelihood of ferric iron reduction and release of dissolved inorganic phosphorus to the water column (as described in Preece et al., 2019). This has the effect of limiting available phosphorus to cyanobacteria and harmful cyanobacteria. Additionally, injection of oxygen into otherwise anoxic waters often has the effect of converting ammonia to nitrate (as described in Preece et al., 2019). It is further possible that hypolimnetic oxygenation increases availability of nitrate to denitrifying bacteria, which may increase denitrification rates and removal of nitrogen from waterbodies (as described in Preece et al., 2019).

The effectiveness of hypolimnetic oxygenation in measurably decreasing internal nutrient loading is dependent on a number of factors including the ability of the technique to achieve at least 2 mg dissolved oxygen/L at the sediment water interface, sediment hydroxide-oxide (e.g., iron and aluminum species) concentrations, and sulfur concentrations (Preece et al. 2019). Furthermore, without external nutrient controls, long-term hypolimnetic oxygenation is necessary to minimize internal nutrient loading (as described in Preece et al., 2019).

(Preece et al. 2019) determined that when the criteria described above are met, hypolimnetic oxygenation is effective in decreasing phosphorus and ammonia concentrations in lakes and reservoirs. Furthermore, there are numerous case studies reporting a decrease in cyanobacteria and/or algal biomass, and/or chlorophyll-a concentrations, concurrent with implementation of hypolimnetic oxygenation (Kibuye et al. 2021a; Preece et al. 2019). Bormans et al. (2016) also reviewed strategies for suppressing internal P loading using hypolimnetic oxygenation while maintaining thermal stratification.

Hypolimnetic oxygenation is a relatively expensive cyanobacteria and harmful algae remediation technique, with annual operation costs potentially greater than $2 million (adjusted to 2022 $US) and similar initial capital investments (Beutel, 2002). Indeed, the technique may not be cost effective in very large lakes. Finally, hypolimnetic oxygenation may have the unintended consequence of increasing water temperatures, particularly if system operation erodes the metalimnion. Kibuye et al. (2021a) summarized several studies reporting water temperature increases ranging from 0.5 – 9 °C during hypolimnetic oxygenation.

<u>Light manipulation</u>

Light is critical for all phytoplankton as they utilize it for photosynthesis. Light reduction management strategies can be used to block sunlight from reaching cyanobacteria to suppress growth rates and shift the competitive advantage to other phytoplankton species (Visser et al. 2016). However, physically blocking light can inhibit photosynthesis by all phytoplankton, which can have profound food web and ecosystem consequences. Light manipulation can be achieved through the use of light blocking dyes, glacial rock powder, solar panels, or other light blocking mechanisms (Gaskill et al. 2020). These light filtering techniques are most effective in small lakes and ponds (ITRC, 2020).

One of the most commonly marketed dyes is Aquashade, which is comprised of the food-colorant dyes, Acid Blue 9 (erioglaucine; CAS Registry Number 3844-45-9) and Acid Yellow 23 (tartrazine; CAS Registry Number 1934-21-0). The dye works by restricting light penetration primarily at wavelengths of 600–650 nm, thus reducing light available for photosynthesis. The utility of dye products to control cyanobacteria has shown mixed effects and there is little in the published literature demonstrating their effectiveness (Boyd et al. 1982; Tucker & Mischke, 2020). Nevertheless, these dyes are

commonly added to small waterbodies. This is because the dyes are low cost, require little technical expertise or equipment, and have low potential for adverse ecological impacts. After the initial dose, it is necessary to continue adding additional dye to maintain the light filtering properties (Ludwig et al., 2020).

Glacial rock powder has shown promise in reducing cyanobacteria biomass, but to date this technique has been limited to laboratory mesocosms. Gaskill et al. (2020) found that glacial rock powder reduced light availability and caused cryptophytes to replace cyanobacteria. The researchers suggest that when nutrients are replete, cryptophytes can outcompete cyanophytes for light. However, the researchers caution that further experimental work is necessary before this technique can be recommended as a light mitigation strategy.

Floating solar panels have been increasingly used as an alternative to land based solar panels. Deploying floating solar panels enhances the performance and electricity generation compared to ground-based panels (Oliveira-Pinto & Stokkermans, 2020). Although solar panels are not installed directly for cyanobacteria mitigation, a growing number of studies have begun evaluating how the technology impacts cyanobacteria populations (Exley et al. 2022). Floating solar panels have been shown to cool water temperatures and block light to slow phytoplankton growth depending on the location of the floating panels as well as surface coverage (Exley et al. 2022). Modeling completed by Exley et al. (2022) showed cyanobacteria dominance did not increase with increasing floating solar panel coverage for any of the deployment scenarios considered. However, partial surface shading with solar panels could increase shade-tolerant or lower-optimum temperature cyanobacteria species by decreasing macrophyte coverage (Yamamichi et al., 2018). Further study is necessary to fully elucidate the impacts of solar panels on phytoplankton communities.

Collectively, light manipulation strategies may be useful in some water bodies to control cyanobacteria, but the technique is generally considered to be experimental. Furthermore, this technique will only be applicable is certain water bodies and the impacts of physically filtering sunlight on the ecology of a waterbody warrants further research.

<u>Sonication</u>

Sonication (also termed ultrasound and/or ultrasonication) inhibits growth and induces mortality in cyanobacteria by disrupting the function of gas vesicles in cyanobacteria cells, resulting in a loss of buoyancy control and disaggregation from colonial or filamentous cell clusters (Ahn et al. 2003; as summarized in Leclercq et al. 2014; Vaughan et al. 2023). This manner of disruption is dependent on factors such as cell wall size and strength, so use of a specific sonic frequency allows targeted treatment of specific cyanobacteria species with limited effect to non-target taxa, and virtually no effect to organisms lacking gas vesicles (as summarized in Vaughan et al., 2023). Secondarily,

sonication may reduce cyanobacteria photosynthetic potential via damage to the cyanobacteria-specific photosynthetic pigment phycocyanin (Ahn et al., 2003; as summarized in Vaughan et al., 2023). Sonication devices are available commercially (e.g., the MPC-Buoy from LG Sonic), and are typically deployed with a buoy and small solar array (Vaughan et al. 2023); multiple transponders are necessary for all but the smallest bodies of water in order to achieve the proper sonication "dose." Most studies use frequencies ranging from 22 to 50 kHz (as summarized in Kibuye et al. 2021b). There are currently no recommended guidelines for effective frequency, power, and exposure time in field settings (Kibuye et al. 2021b).

The majority of sonication assessments are laboratory studies (e.g., those summarized in Lurling et al., 2016 and Rajasekhar et al., 2012; Wu et al., 2011), and uncertainty remains in the literature regarding the effectiveness of sonication in reducing the prevalence and magnitude of cyanobacteria blooms in the field (Kibuye et al. 2021b; (e.g., those summarized in Lurling et al., 2016; Rajasekhar et al. 2012). Indeed, the majority of available field studies (Vaughan et al. 2023 and those summarized therein) report no measurable difference in cyanobacteria biovolume and community composition following the use of sonication. Conversely, (Vaughan et al. 2023) highlight that sonication reduced the need for chemical treatments in drinking water reservoirs and wastewater balance tanks, indicating this technique was effective to some extent in decreasing cyanobacteria biovolume in the field. Ahn et al. (2003) reported that sonication (at 630 W and 22 kHz) appears to have resulted in increased water temperature (by approximately 2 °C), and decreased pH (by approximately 1 pH unit), chlorophyll-*a* (by approximately 75 ug/L), and dissolved oxygen concentrations (by approximately 4 mg/L) in a series of 0.6 m-diameter mesocosms; these findings suggest sonication resulted in cell destruction and an associated reduction in photosynthetic activity over the 10-day study period. However, a concurrent lab study indicated that once sonication ceased, *Microcystis aeruginosa* growth rates recovered quickly, suggesting the need for frequent and repeated sonication treatments to effectively control cyanobacteria in surface waters. This may be mitigated to some extent by implementing sonication treatment in the evening, shortly after *Microcystis aeruginosa* cell division occurs (Ahn et al. 2003). Regardless, given the general paucity of sonication field assessments, and uncertainty regarding the effectiveness of this technique as reported in the limited number of field assessments available, sonication remains an experimental technique requiring additional study. In the limited field studies available, there is also a lack of clarity around operational parameters and treatment longevity, further adding to uncertainty regarding sonication (Kibuye et al., 2021b).

A high degree of uncertainty around the effectiveness of sonication in remediating cyanobacteria blooms means it is difficult to provide specific information regarding labor and costs associated with this technique. It is important to note that several studies (as reported in Vaughan et al. 2023) describe technical difficulties and

equipment failures associated with commercially-available sonication devices deployed in the field.

Additional considerations for this technique include the potential for toxin release from lysed cells (Vaughan et al. 2023), increased water temperatures (Ahn et al. 2003) during treatment, effects to zooplankton and fish, and lake conditions that may impact the effectiveness of sonication. It is possible that high-frequency sonication (e.g., 640 kHz) may be effective in degrading microcystin lysed from cells following treatment (Song et al. 2005), but this effect was observed in a laboratory setting and would likely be very difficult to reproduce in the field given rapid attenuation of sonic waves in natural settings (Rajasekhar et al. 2012). Specifically, sonication is likely less effective in shallow water since light penetration in this circumstance may allow cyanobacteria to photosynthesize after settling in the sediments, rebuild gas vesicles during that time, and eventually migrate back to ideal water depth for photosynthesis (Ahn et al. 2003). Additionally, turbid conditions (e.g., that observed during cyanobacteria blooms) likely reduce the range of sonication (as summarized in Vaughan et al. 2023), requiring additional transducers to achieve an effective sonication dose. Finally, there is some evidence that sonication within the range typically employed in laboratory and field studies leads to rapid mortality in *Daphnia*, and can cause damage to fish skin (as summarized in Lurling et al. 2016 and Kibuye et al. 2021b).

References

Adey, W. H., Kangas, P. C., & Mulbry, W. (2011). Algal Turf Scrubbing: Cleaning Surface Waters with Solar Energy while Producing a Biofuel. *BioScience*, *61*(6), 434–441. https://doi.org/10.1525/bio.2011.61.6.5

Adey, W. H., Laughinghouse, H. D., Miller, J. B., Hayek, L.-A. C., Thompson, J. G., Bertman, S., Hampel, K., & Puvanendran, S. (2013). Algal turf scrubber (ATS) floways on the Great Wicomico River, Chesapeake Bay: Productivity, algal community structure, substrate and chemistry(1). *Journal of Phycology*, *49*(3), 489–501. https://doi.org/10.1111/jpy.12056

Ahn, C.-Y., Park, M.-H., Joung, S.-H., Kim, H.-S., Jang, K.-Y., & Oh, H.-M. (2003). Growth inhibition of Cyanobacteria by ultrasonic radiation: Laboratory and enclosure studies. *Environmental Science & Technology*, *37*(13), 3031–3037. https://doi.org/10.1021/es034048z

Amorim, C.A., Moura, A.N., 2020. Effects of the manipulation of submerged macrophytes, large zooplankton, and nutrients on a cyanobacterial bloom: A mesocosm study in a tropical shallow reservoir. Environ. Pollut. 265, 114997 https://doi.org/10.1016/j.envpol.2020.114997.

Amorim, C.A., Valença, C.R., de Moura-Falc˜ao, R.H., do Nascimento Moura, A., 2019. Seasonal variations of morpho-functional phytoplankton groups influence the topdown control of a cladoceran in a tropical hypereutrophic lake. Aquat. Ecol. 53, 453–464. https://doi.org/10.1007/s10452-019-09701-8.

Barrington, D. J., Reichwaldt, E. S., & Ghadouani, A. (2013). The use of hydrogen peroxide to remove cyanobacteria and microcystins from waste stabilization ponds and hypereutrophic systems. *Ecological Engineering*, *50*, 86–94. https://doi.org/10.1016/j.ecoleng.2012.04.024

Becker, A., Herschel, A., & Wilhelm, C. (2006). Biological Effects of Incomplete Destratification of Hypertrophic Freshwater Reservoir. *Hydrobiologia*, *559*, 85–100. https://doi.org/10.1007/s10750-005-4428-3

Benndorf, J., Böing, W., Koop, J., Neubauer, I., 2002. Top-down control of phyto- plankton: the role of time scale, lake depth and trophic state. Freshw. Biol. 47, 2282–2295.

Beutel, D. M. (2002). Improving raw water quality with hypolimnetic oxygenation. *Proceedings of the American Water Works Association 2002 Annual Meeting*. AWWA 2002 Annual Conference. https://eco2tech.com/wp-content/uploads/Improving-Raw-Water-Quality-with-Hypolimnetic-Oxygenation.pdf

Block, C., & Adam, J. (2022). *Sweetwater Reservoir Aeration/Destratification System* [Final Preliminary Design Technical Memorandum]. Hazen. https://www.sweetwater.org/DocumentCenter/View/3250/Agenda-Item-10---Attachment-2#:~:text=3.1%20Reservoir%20Aeration%2FDestratification%20System%20Objectives,-In%20the%20previous&text=A%20destratification%20system%20destratifies%20a,downwelling%20of%20oxygenated%20surface%20water.

Bormans, M., Maršálek, B., & Jančula, D. (2016). Controlling internal phosphorus loading in lakes by physical methods to reduce cyanobacterial blooms: A review. *Aquatic Ecology*, *50*(3), 407–422. https://doi.org/10.1007/s10452-015-9564-x

Boyd, C. E., Hanapi, M., & Noor, M. (1982). Aquashade(R) Treatment of Channel Catfish Ponds. *North American Journal of Fisheries Management*, *2*(2), 193–196. https://doi.org/10.1577/1548-8659(1982)2<193:ATOCCP>2.0.CO;2

Boylan, J., & Morris, J. (2003). Limited Effects of Barley Straw on Algae and Zooplankton in a Midwestern Pond. *Lake and Reservoir Management*, *19*(3), 265–271. https://doi.org/10.1080/07438140309354091

Buley, R. P., Adams, C., Belfiore, A. P., Fernandez-Figueroa, E. G., Gladfelter, M. F., Garner, B., & Wilson, A. E. (2021). Field evaluation of seven products to control cyanobacterial blooms in aquaculture.

Environmental Science and Pollution Research International, *28*(23), 29971–29983. https://doi.org/10.1007/s11356-021-12708-0

Caffrey, J. M., & Monahan, C. (1999). Filamentous algal control using barley straw. *Hydrobiologia*, *415*(0), 315–318. https://doi.org/10.1023/A:1003884211027

Chen, S., Carey, C. C., Little, J. C., Lofton, M. E., McClure, R. P., & Lei, C. (2018). Effectiveness of a bubble-plume mixing system for managing phytoplankton in lakes and reservoirs. *Ecological Engineering*, *113*, 43–51. https://doi.org/10.1016/j.ecoleng.2018.01.002

Cooke, Welch, Peterson, & Nichols. (2005). *Restoration and Management of Lakes and Reservoirs*. CRC Press. https://www.routledge.com/Restoration-and-Management-of-Lakes-and-Reservoirs/Cooke-Welch-Peterson-Nichols/p/book/9781566706254

Copetti, D., Finsterle, K., Marziali, L., Stefani, F., Tartari, G., Douglas, G., Reitzel, K., Spears, B. M., Winfield, I. J., Crosa, G., D'Haese, P., Yasseri, S., & Lürling, M. (2016). Eutrophication management in surface waters using lanthanum modified bentonite: A review. *Water Research*, *97*, 162–174. https://doi.org/10.1016/j.watres.2015.11.056

Dinkins, K., Zivojnovich, M., Stewart, A., & Bazurto, R. (2009). Review of Large Scale Algal Turf Scrubber® Algae Based Water Treatment Systems and Algal Biomass Production and Use. *Proceedings of the Water Environment Federation*, *7*, 7972–7977. https://doi.org/10.2175/193864709793900078

Douglas, G. B., Hamilton, D. P., Robb, M. S., Pan, G., Spears, B. M., & Lurling, M. (2016). Guiding principles for the development and application of solid-phase phosphorus adsorbents for freshwater ecosystems. *Aquatic Ecology*, *50*(3), 385–405. https://doi.org/10.1007/s10452-016-9575-2

Drábková, M., Admiraal, W., & Maršálek, B. (2007). Combined Exposure to Hydrogen Peroxide and LightSelective Effects on Cyanobacteria, Green Algae, and Diatoms. *Environmental Science & Technology*, *41*(1), 309–314. https://doi.org/10.1021/es060746i

Drenner R.W., Hambright K.D. 1999. Review: biomanipulation of fish assemblages as a lake restoration technique. Arch Hydrobiol 146:129–165.

Exley, G., Page, T., Thackeray, S. J., Folkard, A. M., Couture, R.-M., Hernandez, R. R., Cagle, A. E., Salk, K. R., Clous, L., Whittaker, P., Chipps, M., & Armstrong, A. (2022). Floating solar panels on reservoirs impact phytoplankton populations: A modelling experiment. *Journal of Environmental Management*, *324*, 116410. https://doi.org/10.1016/j.jenvman.2022.116410

Fan, F., Shi, X., Zhang, M., Liu, C., & Chen, K. (2019). Comparison of algal harvest and hydrogen peroxide treatment in mitigating cyanobacterial blooms via an in situ mesocosm experiment. *Science of The Total Environment*, *694*, 133721. https://doi.org/10.1016/j.scitotenv.2019.133721

Ferguson, J. F., Jenkins, D., & Eastman, J. (1973). Calcium Phosphate Precipitation at Slightly Alkaline pH Values. *Journal (Water Pollution Control Federation)*, *45*(4), 620–631.

Ferrier, M. D., Butler, B. R., Terlizzi, D. E., & Lacouture, R. V. (2005). The effects of barley straw (Hordeum vulgare) on the growth of freshwater algae. *Bioresource Technology*, *96*(16), 1788–1795. https://doi.org/10.1016/j.biortech.2005.01.021

Garcia Chance, L. M., Van Brunt, S. C., Majsztrik, J. C., & White, S. A. (2019). Short- and long-term dynamics of nutrient removal in floating treatment wetlands. *Water Research*, *159*, 153–163. https://doi.org/10.1016/j.watres.2019.05.012

Gaskill, J. A., Harris, T. D., & North, R. L. (2020). Phytoplankton Community Response to Changes in Light: Can Glacial Rock Flour Be Used to Control Cyanobacterial Blooms? *Frontiers in Environmental Science*, *8*, 540607. https://doi.org/10.3389/fenvs.2020.540607

Geer, T. D., Kinley, C. M., Iwinski, K. J., Calomeni, A. J., & Rodgers, J. H. (2016). Comparative toxicity of sodium carbonate peroxyhydrate to freshwater organisms. *Ecotoxicology and Environmental Safety*, *132*, 202–211. https://doi.org/10.1016/j.ecoenv.2016.05.037

Gibbs, M. M., Hickey, C. W., & Özkundakci, D. (2011). Sustainability assessment and comparison of efficacy of four P-inactivation agents for managing internal phosphorus loads in lakes: Sediment incubations. *Hydrobiologia*, *658*(1), 253–275. https://doi.org/10.1007/s10750-010-0477-3

Gołdyn, R., Podsiadłowski, S., Dondajewska, R., Kozak, A. (2014). The sustainable restoration of lakes-towards the challenges of the water framework directive. *Ecohydrol. Hydrobiol.* 14, 68–74. https://doi.org/10.1016/j.ecohyd.2013.12.001

Greenfield, D. I., Duquette, A., Goodson, A., Keppler, C. J., Williams, S. H., Brock, L. M., Stackley, K. D., White, D., & Wilde, S. B. (2014). The Effects of Three Chemical Algaecides on Cell Numbers and Toxin Content of the Cyanobacteria Microcystis aeruginosa and Anabaenopsis sp. *Environmental Management*, *54*, 1110–1120. https://doi.org/10.1007/s00267-014-0339-2

Hanson, M. J., & Stefan, H. G. (1984). Side Effects of 58 Years of Copper Sulfate Treatment of the Fairmont Lakes, Minnesota1. *JAWRA Journal of the American Water Resources Association*, *20*, 889–900. https://doi.org/10.1111/j.1752-1688.1984.tb04797.x

Hickey, C. W., & Gibbs, M. M. (2009). Lake sediment phosphorus release management—Decision support and risk assessment framework. *New Zealand Journal of Marine and Freshwater Research*, *43*(3), 819–856. https://doi.org/10.1080/00288330909510043

Huser, B. J., Egemose, S., Harper, H., Hupfer, M., Jensen, H., Pilgrim, K. M., Reitzel, K., Rydin, E., & Futter, M. (2016). Longevity and effectiveness of aluminum addition to reduce sediment phosphorus release and restore lake water quality. *Water Research*, *97*, 122–132. https://doi.org/10.1016/j.watres.2015.06.051

Islami, H. R., & Filizdeh, Y. (2011). Use of barley straw to control nuisance freshwater algae. *Journal of the American Water Works Association*, *103*, 111–118. https://doi.org/10.1002/j.1551-8833.2011.tb11458.x

ITRC (Interstate Technology & Regulatory Council). (2020). *Strategies for Preventing and Managing Harmful Cyanobacterial Blooms (HCB-1).* Washington, D.C.:Interstate Technology & Regulatory Council, HCB Team. www.itrcweb.org.

Jeppesen, E., Søndergaard, M., Lauridsen, T.L., Davidson, T.A., Liu, Z., Mazzeo, N., Trochine, C., Özkan, K., Jensen, H.S., Trolle, D. and Starling, F., 2012. Biomanipulation as a restoration tool to combat eutrophication: recent advances and future challenges. *Advances in ecological research*, *47*, pp.411-488.

Jing, L., Bai, S., Li, Y., Peng, Y., Wu, C., Liu, J., Liu, G., Xie, Z., & Yu, G. (2019). Dredging project caused short-term positive effects on lake ecosystem health: A five-year follow-up study at the integrated lake ecosystem level. *The Science of the Total Environment*, *686*, 753–763. https://doi.org/10.1016/j.scitotenv.2019.05.133

Jungo, E., Visser, P. M., Stroom, J., & Mur, L. R. (2001). Artificial mixing to reduce growth of the blue-green alga Microcystis in Lake Nieuwe Meer, Amsterdam: An evaluation of 7 years of experience. *Water Supply*, *1*(1), 17–23. https://doi.org/10.2166/ws.2001.0003

Kiani, M., Tammeorg, P., Niemistö, J., Simojoki, A., & Tammeorg, O. (2020). Internal phosphorus loading in a small shallow Lake: Response after sediment removal. *Science of The Total Environment*, *725*, 138279. https://doi.org/10.1016/j.scitotenv.2020.138279

Kibuye, F. A., Zamyadi, A., & Wert, E. C. (2021a). A critical review on operation and performance of source water control strategies for cyanobacterial blooms: Part I-chemical control methods. *Harmful Algae*, *109*, 102099. https://doi.org/10.1016/j.hal.2021.102099

Kibuye, F. A., Zamyadi, A., & Wert, E. C. (2021b). A critical review on operation and performance of source water control strategies for cyanobacterial blooms: Part II-mechanical and biological control methods. *Harmful Algae*, *109*, 102119. https://doi.org/10.1016/j.hal.2021.102119

Kinley-Baird, C., Calomeni, A., Berthold, D. E., Lefler, F. W., Barbosa, M., Rodgers, J. H., & Laughinghouse, H. D. (2021). Laboratory-scale evaluation of algaecide effectiveness for control of microcystin-producing cyanobacteria from Lake Okeechobee, Florida (USA). *Ecotoxicology and Environmental Safety*, *207*, 111233. https://doi.org/10.1016/j.ecoenv.2020.111233

Kleeberg, A., Herzog, C., Hupfer, M., 2013. Redox sensitivity of iron in phosphorus binding does not impede lake restoration. Water Res. 47, 1491–1502. https://doi. org/10.1016/j.watres.2012.12.014.

Leclercq, D. J. J., Howard, C. Q., Hobson, P., Dickson, S., Zander, A. C., & Burch, M. (2014). CONTROLLING CYANOBACTERIA WITH ULTRASOUND. *Proceedings of the 43rd International Congress on Noise Control Engineering*. Inter-noise 2014.

Lefler, F. W., Berthold, D. E., Barbosa, M., & Laughinghouse, H. D., IV. (2022). The Effects of Algaecides and Herbicides on a Nuisance Microcystis wesenbergii-Dominated Bloom. *Water*, *14*(11), 1739.

Lentsch, L. D., C. W. Thompson, and R. L. Spateholts. 2001. Overview of a large-scale chemical treatment success story: Strawberry Valley, Utah. Pages 63–79 *in* R. L. Cailteux, L. DeMong, B. J. Finlayson, W. Horton, W. McClay, R. A. Schnick, and C. Thompson, editors. *Rotenone in fisheries: are the rewards worth the risks?* American Fisheries Society, Bethesda, Maryland.

Leoni, B., Morabito, G., Rogora, M., Pollastro, D., Mosello, R., Arisci, S., Forasacco, E., Garibaldi, L. (2007). Response of planktonic communities to calcium hydroxide addition in a hardwater eutrophic lake: results from a mesocosm experiment. *Limnology* 8, 121–130. https://doi.org/10.1007/s10201-007-0202-8.

Liu, K., Jiang, L., Yang, J., Ma, S., Chen, K., Zhang, Y., & Shi, X. (2022). Comparison of three flocculants for heavy cyanobacterial bloom mitigation and subsequent environmental impact. *Journal of Oceanology and Limnology*, *40*(5), 1764–1773. https://doi.org/10.1007/s00343-022-1351-7

Ludwig, G. M., Kang, Mucci, van Oosterhout, F., Noyma, N. P., Mirand, M., Huszar, V. L., Waajen, G., & Marinho, M. M. (2020). The Effect of the Dye Aquashade® on Water Quality, Phytoplankton, Zooplankton, and Sunshine Bass, Morone chrysops×M. saxatilis, Fingerling Production in Fertilized Culture Ponds—Ludwig—2010—Journal of the World Aquaculture Society—Wiley Online Library. *Journal of the World Aquaculture Society*, *41*, 40–48.

Lürling, M., Kang, L., Mucci, M., van Oosterhout, F., Noyma, N. P., Miranda, M., Huszar, V. L. M., Waajen, G., & Marinho, M. M. (2020). Coagulation and precipitation of cyanobacterial blooms. *Ecological Engineering*, *158*, 106032. https://doi.org/10.1016/j.ecoleng.2020.106032

Lürling, M., Waajen , G., de Senerpont Domsis, L. N. (2016). Evaluation of several end-of-pipe measures proposed to control cyanobacteria. *Aquatic Ecology, 50, 499-519*.

Lusty, M. W., & Gobler, C. J. (2020). Toxins | Free Full-Text | The Efficacy of Hydrogen Peroxide in Mitigating Cyanobacterial Blooms and Altering Microbial Communities across Four Lakes in NY, USA. *Toxins*, *12*(7), 428. https://doi.org/10.3390/toxins12070428

Lynch, J., Fox, L. J., Owen Jr., J. S., & Sample, D. J. (2015). Evaluation of commercial floating treatment wetland technologies for nutrient remediation of stormwater. *Ecological Engineering*, *75*, 61–69. https://doi.org/10.1016/j.ecoleng.2014.11.001

Lyon, S., Horne, A., Jordahl, J., Emond, H., & Carlson, K. (2009). *Preliminary Feasibility Assessment of Constructed Treatment Wetlands in the Vicinity of the Klamath Hydroelectric Project*. Prepared by CH2M HILL and Alex Horne Associates. Prepared for PacifiCorps Energy. https://www.pacificorp.com/content/dam/pcorp/documents/en/pacificorp/energy/hydro/klamath-river/water-quality-reports-and-

data/reports/Klamath_Preliminary_Feasibility_Assessment_of_Constucted_Treatment_Wetlands.pdf

Ma, J., Tong, S., Wang, P., & Chen, J. (2010). Toxicity of Seven Herbicides to the Three Cyanobacteria Anabaena flos-aquae, Microcystis flos-aquae and Mirocystis aeruginosa. *International Journal of Environmental Research*, *4*(2), 347–352.

Magnusson, M., Heimann, K., Quayle, P., & Negri, A. P. (2010). Additive toxicity of herbicide mixtures and comparative sensitivity of tropical benthic microalgae. *Marine Pollution Bulletin*, *60*(11), 1978–1987. https://doi.org/10.1016/j.marpolbul.2010.07.031

Masters, B. (2012). The ability of vegetated floating Islands to improve water quality in natural and constructed wetlands: A review. *Water Practice and Technology*, *7*(1). https://doi.org/10.2166/wpt.2012.022

Matthiljs, H. C. P., Jancula, D., Visser, P. M., Marsalek, B. (2016). Existing and emerging cyanocidal compounds: new perspectives for cyanobacterial bloom mitigation. Aquatic Ecology, 50, 443-460.

Mehner, T., Diekmann, M., Gonsiorczyk, T., Kasprzak, P., Koschel, R., Krienitz, L., Rumpf, M., Schulz, M., Wauer, G. (2008). Rapid recovery from eutrophication of a stratified lake by disruption of internal nutrient load. *Ecosystems* 11, 1142–1156. https://doi.org/10.1007/s10021-008-9185-5.

Messager, M. L., Lehner, B., Grill, G., Nedeva, I., & Schmitt, O. (2016). Estimating the volume and age of water stored in global lakes using a geo-statistical approach. *Nature Communications*, *7*(1), Article 1. https://doi.org/10.1038/ncomms13603

Moore, M. V., & Winner, R. W. (1989). Relative sensitivity of Cerodaphnia dubia laboratory tests and pond communities of zooplankton and benthos to chronic copper stress. *Aquatic Toxicology*, *15*(4), 311–330. https://doi.org/10.1016/0166-445X(89)90044-1

Mucci, M., Guedes, I. A., Faassen, E. J., & Lürling, M. (2020). Chitosan as a Coagulant to Remove Cyanobacteria Can Cause Microcystin Release. *Toxins*, *12*(11), Article 11. https://doi.org/10.3390/toxins12110711

Mulbry, W., Kangas, P., & Kondrad, S. (2010). Toward scrubbing the bay: Nutrient removal using small algal turf scrubbers on Chesapeake Bay tributaries. *Ecological Engineering*, *36*(4), 536–541. https://doi.org/10.1016/j.ecoleng.2009.11.026

Oliveira-Pinto, S., & Stokkermans, J. (2020). Assessment of the potential of different floating solar technologies – Overview and analysis of different case studies. *Energy Conversion and Management*, *211*, 112747. https://doi.org/10.1016/j.enconman.2020.112747

Olsson, F., Mackay, E. B., Barker, P., Davies, S., Hall, R., Spears, B., Exley, G., Thackeray, S. J., & Jones, I. D. (2022). Can reductions in water residence time be used to disrupt seasonal stratification and control internal loading in a eutrophic monomictic lake? *Journal of Environmental Management*, *304*, 114–169. https://doi.org/10.1016/j.jenvman.2021.114169

Paerl, H. W., & Barnard, M. A. (2020). Mitigating the global expansion of harmful cyanobacterial blooms: Moving targets in a human- and climatically-altered world. *Harmful Algae*, *96*, 101845. https://doi.org/10.1016/j.hal.2020.101845

Paerl, H. W., & Otten, T. G. (2013a). Blooms Bite the Hand That Feeds Them. *Science*, *342*(6157), 433–434. https://doi.org/10.1126/science.1245276

Pan, G., Zhang, M.-M., Chen, H., Zou, H., & Yan, H. (2006a). Removal of cyanobacterial blooms in Taihu Lake using local soils. I. Equilibrium and kinetic screening on the flocculation of Microcystis aeruginosa using commercially available clays and minerals. *Environmental Pollution*, *141*(2), 195–200. https://doi.org/10.1016/j.envpol.2005.08.041

Pan, G., Zou, H., Chen, H., & Yuan, X. (2006b). Removal of harmful cyanobacterial blooms in Taihu Lake using local soils III. Factors affecting the removal efficiency and an in situ field experiment using

chitosan-modified local soils. *Environmental Pollution*, *141*(2), 206–212. https://doi.org/10.1016/j.envpol.2005.08.047

Papadimitriou, T., Katsiapi, M., Stefanidou, N., Paxinou, A., Poulimenakou, V., Laspidou, C. S., Moustaka-Gouni, M., & Kormas, K. A. (2022). Differential Effect of Hydroxen Peroxide on Toxic Cyanobacteria of Hypertrophic Mediterranean Waterbodies. *Sustainability*, *14*(1), Article 1. https://doi.org/10.3390/su14010123

Pavlineri, N., Skoulikidis, N. Th., & Tsihrintzis, V. A. (2017). Constructed Floating Wetlands: A review of research, design, operation and management aspects, and data meta-analysis. *Chemical Engineering Journal*, *308*, 1120–1132. https://doi.org/10.1016/j.cej.2016.09.140

Peng, L., Lei, L., Xiao, L., & Han, B. (2019). Cyanobacterial removal by a red soil-based flocculant and its effect on zooplankton: An experiment with deep enclosures in a tropical reservoir in China. *Environmental Science and Pollution Research*, *26*(30), 30663–30674. https://doi.org/10.1007/s11356-018-2572-3

Peng, G., Zhou, X., Xie, B., Huang, C., Uddin, M.M., Chen, X. and Huang, L., 2021. Ecosystem stability and water quality improvement in a eutrophic shallow lake via long-term integrated biomanipulation in Southeast China. *Ecological Engineering*, *159*, p.106119.

Piel, T., Sandrini, G., White, E., Xu, T., Schuurmans, J. M., Huisman, J., & Visser, P. M. (2019). Suppressing Cyanobacteria with Hydrogen Peroxide Is More Effective at High Light Intensities. *Toxins*, *12*(1), 18. https://doi.org/10.3390/toxins12010018

Pillinger, J. M., Cooper, J. A., & Ridge, I. (1994). Role of phenolic compounds in the antialgal activity of barley straw. *Journal of Chemical Ecology*, *20*, 1557–1569. https://doi.org/10.1007/BF02059880

Preece, E. P., Moore, B. C., Skinner, M. M., Child, A., & Dent, S. (2019). A review of the biological and chemical effects of hypolimnetic oxygenation. *Lake and Reservoir Management*, *35*(3), 229–246. https://doi.org/10.1080/10402381.2019.1580325

Quaak, M., van der Does, J., Boers, P., van der Vlugt, J. (1993). A new technique to reduce internal phosphorus loading by in-lake phosphate fixation in shallow lakes. *Hydrobiologia* 253, 337–344. https://doi.org/10.1007/BF00050759.

Rajasekhar, P., Fan, L., Nguyen, T., & Roddick, F. A. (2012). A review of the use of sonication to control cyanobacterial blooms. *Water Research*, *46*(14), 4319–4329. https://doi.org/10.1016/j.watres.2012.05.054

Ralston, D. K., Brosnahan, M. L., Fox, S. E., Lee, K. D., & Anderson, D. M. (2015). Temperature and Residence Time Controls on an Estuarine Harmful Algal Bloom: Modeling Hydrodynamics and Alexandrium fundyense in Nauset Estuary. *Estuaries and Coasts*, *38*(6), 2240–2258. https://doi.org/10.1007/s12237-015-9949-z

Raman, R., & Cook, B. (1988). *Guildelines for applying copper sulfate as an algicide: Lake Loami field study* (ISWS Contract Report CR 450 ILENR/RE-WR-88/19). Prepared from the Illinois Department of Energy and Natural Resources, Office of Research and Planning by Illinois State Water Survey, Water Quality Section: Peoria, IL. http://hdl.handle.net/2142/74800

Reichwaldt, E. S., Zheng, L., Barrington, D. J., & Ghadouani, A. (2012). Acute Toxicological Response of *Daphnia* and *Moina* to Hydrogen Peroxide. *Journal of Environmental Engineering*, *138*(5), 607–611. https://doi.org/10.1061/(ASCE)EE.1943-7870.0000508

Riza, M., Ehsan, M. N., Pervez, M. N., Khyum, M. M. O., Cai, Y., & Naddeo, V. (2023). Control of eutrophication in aquatic ecosystems by sustainable dredging: Effectiveness, environmental impacts, and implications. *Case Studies in Chemical and Environmental Engineering*, *7*, 100297. https://doi.org/10.1016/j.cscee.2023.100297

Romo, S., Soria, J., Fernández, F., Ouahid, Y., & Barón-Solá, Á. (2013). Water residence time and the dynamics of toxic cyanobacteria. *Freshwater Biology*, *58*(3), 513–522. https://doi.org/10.1111/j.1365-2427.2012.02734.x

Ross, G., Haghseresht, F., & Cloete, T. E. (2008). The effect of pH and anoxia on the performance of Phoslock®, a phosphorus binding clay. *Harmful Algae*, *7*(4), 545–550. https://doi.org/10.1016/j.hal.2007.12.007

Sample, D. J., Wang, C.-Y., & Fox, L. J. (2013). *Innovative Best Management Fact Sheet No. 1: Floating Treatment Wetlands*. https://vtechworks.lib.vt.edu/server/api/core/bitstreams/7b74d251-5d5d-45a0-9428-5961406ac11f/content

Sandrini, G., Piel, T., Xu, T., White, E., Qin, H., Slot, P. C., Huisman, J., & Visser, P. M. (2020). Sensitivity to hydrogen peroxide of the bloom-forming cyanobacterium Microcystis PCC 7806 depends on nutrient availability. *Harmful Algae*, *99*, 101916. https://doi.org/10.1016/j.hal.2020.101916

Seelos, M., Beutel, M., Austin, C. M., Wilkinson, E., & Leal, C. (2021). Effects of hypolimnetic oxygenation on fish tissue mercury in reservoirs near the new Almaden Mining District, California, USA. *Environmental Pollution (Barking, Essex: 1987)*, *268*(Pt A), 115759. https://doi.org/10.1016/j.envpol.2020.115759

Scharf, W., 2007. Biomanipulation as a useful water quality management tool in deep stratifying reservoirs. *Hydrobiologia*, *583*(1), pp.21-42.

Skinner, M. (2020). *Feasibility of using floating wetlands to treat Upper Klamath Lake phosphorus load.* U.S. Fish and Wildlife Servic.

Song, W., Teshiba, T., Rein, K., & O'Shea, K. E. (2005). Ultrasonically Induced Degradation and Detoxification of Microcystin-LR (Cyanobacterial Toxin). *Environmental Science & Technology*, *39*(16), 6300–6305. https://doi.org/10.1021/es048350z

Spangler, J. T., Sample, D. J., Fox, L. J., Owen, J. S., & White, S. A. (2019). Floating treatment wetland aided nutrient removal from agricultural runoff using two wetland species. *Ecological Engineering*, *127*, 468–479. https://doi.org/10.1016/j.ecoleng.2018.12.017

Spoof, L., Jaakkola, S., Važić, T., Häggqvist, K., Kirkkala, T., Ventelä, A.-M., Kirkkala, T., Svirčev, Z., & Meriluoto, J. (2020). Elimination of cyanobacteria and microcystins in irrigation water—Effects of hydrogen peroxide treatment. *Environmental Science and Pollution Research*, *27*(8), 8638–8652. https://doi.org/10.1007/s11356-019-07476-x

Stewart, F. (2007). *Biomimetic floating islands that maximize plant and microbial synergistic relationships to revitalize degraded fisheries, wildlife habitats, and human water resources*. Floating Islands International for the Montana Board of Research and Commercialization Technology. https://midwestfloatingisland.com/wp-content/uploads/2015/05/2007-Final-Report-to-Montana-Board-of-Research-and-Commercialization-Technology.pdf

Stewart, F. M., Mulholland, T., Cunningham, A. B., Kania, B. G., & Osterlund, M. T. (2008). Floating islands as an alternative to constructed wetlands for treatment of excess nutrients from agricultural and municipal wastes—Results of laboratory-scale tests. *Land Contamination & Reclamation*, *16*(1), 25–33. https://doi.org/10.2462/09670513.874

Stroom, J. M., & Kardinaal, W. E. A. (2016). How to combat cyanobacterial blooms: Strategy toward preventive lake restoration and reactive control measures. *Aquatic Ecology*, *50*, 541–576. https://doi.org/10.1007/s10452-016-9593-0

Sukenik, A., & Kaplan, A. (2021). Microorganisms | Free Full-Text | Cyanobacterial Harmful Algal Blooms in Aquatic Ecosystems: A Comprehensive Outlook on Current and Emerging Mitigation and Control Approaches. *Microorganisms*, *9*(7), 1472. https://doi.org/10.3390/microorganisms9071472

Teixeira, M. R., Rosa, M. J., Sorlini, S., Biasibetti, M., Christophoridis, C., & Edwards, C. (2020). Removal of Cyanobacteria and Cyanotoxins by Conventional Physical-chemical Treatment. In *Water Treatment for Purification from Cyanobacteria and Cyanotoxins* (pp. 69–97). John Wiley & Sons, Ltd. https://doi.org/10.1002/9781118928677.ch3

Triest, L., Stiers, I. Van Onsem, S., 2016. Biomanipulation as a nature-based solution to reduce cyanobacterial blooms. *Aquatic ecology*, *50*, 461-483.

Tucker, C. S., & Mischke, C. C. (2020). The Pond Dye, Aquashade, Does Not Prevent Cyanobacterial Off-Flavors in Pond-Grown Channel Catfish. *North American Journal of Aquaculture*, *82*(1), 101–107. https://doi.org/10.1002/naaq.10128

Vašek, M., Prchalová, M., Peterka, J., Ketelaars, H.A., Wagenvoort, A.J., Čech, M., Draštík, V., Říha, M., Jůza, T., Kratochvíl, M. and Mrkvička, T., 2013. The utility of predatory fish in biomanipulation of deep reservoirs. *Ecological Engineering*, *52*, pp.104-111.

Vaughan, L., Barnett, D., Bourke, E., Burrows, H., Robertson, F., Smith, B., Cashmore, J., Welk, M., Burch, M., & Zamyadi, A. (2023). Evaluating Ultrasonicator Performance for Cyanobacteria Management at Freshwater Sources. *Toxins*, *15*(3), Article 3. https://doi.org/10.3390/toxins15030186

Visser, P. M., Ibelings, B. W., Bormans, M., & Huisman, J. (2016). Artificial mixing to control cyanobacterial blooms: A review. *Aquatic Ecology*, *50*(3), 423–441. https://doi.org/10.1007/s10452-015-9537-0

Watercourse Engineering. (2013). *2012 Localized Treatment of Copco Cove in Copco Reservoir Using Environmentally Safe Algaecide*. Prepared for PacifiCorp Energy by Watercourse Engineering: Davis, CA. https://www.pacificorp.com/content/dam/pcorp/documents/en/pacificorp/energy/hydro/klamath-river/khsa-implementation/technical-documents/2012%20Final%20Algaecide%20Tech%20Report%20(7-24-13)-P8.pdf

Weenink, E. F. J., Matthijs, H. C. P., Schuurmans, J. M., Piel, T., van Herk, M. J., Sigon, C. A. M., Visser, P. M., & Huisman, J. (2021). Interspecific protection against oxidative stress: Green algae protect

harmful cyanobacteria against hydrogen peroxide. *Environmental Microbiology*, *23*(5), 2404–2419. https://doi.org/10.1111/1462-2920.15429

Welch, E. B., & Cooke, G. D. (1999). Effectiveness and Longevity of Phosphorus Inactivation with Alum. *Lake and Reservoir Management*, *15*(1), 5–27. https://doi.org/10.1080/07438149909353948

White, S. A., & Cousins, M. M. (2013). Floating treatment wetland aided remediation of nitrogen and phosphorus from simulated stormwater runoff. *Ecological Engineering*, *61*(Part A), 207–215. https://doi.org/10.1016/j.ecoleng.2013.09.020

White, S., Seda, B., Cousins, M., Klaine, S., & Whitwell, T. (2009). Nutrient remediation using vegetated floating mats. *Proceedings of of the Annual Research Conference of the Southern Nursery Association*, *54*, 39–43. https://www.researchgate.net/profile/Sarah-White-37/publication/290163443_Nutrient_remediation_using_vegetated_floating_mats/links/56994ad508aea147694335f3/Nutrient-remediation-using-vegetated-floating-mats.pdf

Williams, M. C. W., Conroy, J. D., Miner, J. G., & Farver, J. R. (2015). Indirect Effects of Copper Sulfate Addition on Zooplankton Communities in Ohio Upground Reservoirs. *The Ohio Journal of Science*, *115*(2), Article 2. https://doi.org/10.18061/ojs.v115i2.4638

Wu, X., Joyce, E. M., & Mason, T. J. (2011). The effects of ultrasound on cyanobacteria. *Harmful Algae*, *10*(6), 738–743. https://doi.org/10.1016/j.hal.2011.06.005

Yamamichi, M., Kazama, T., Tokita, K., Katano, I., Doi, H., Yoshida, T., Hairston, N. G., & Urabe, J. (2018). A shady phytoplankton paradox: When phytoplankton increases under low light. *Proceedings of the Royal Society B: Biological Sciences*, *285*(1882), 20181067. https://doi.org/10.1098/rspb.2018.1067

Zamparas, M., & Zacharias, I. (2014). Restoration of eutrophic freshwater by managing internal nutrient loads. A review. *Science of The Total Environment*, *496*, 551–562. https://doi.org/10.1016/j.scitotenv.2014.07.076

Zeng, J., Yang, L., & Wang, W.-X. (2010). High sensitivity of cyanobacterium Microcystis aeruginosa to copper and the prediction of copper toxicity. *Environmental Toxicology and Chemistry*, *29*(10), 2260–2268. https://doi.org/10.1002/etc.266

Zhang, D. Q., Jinadasa, K. B. S. N., Gersberg, R. M., Liu, Y., Ng, W. J., & Tan, S. K. (2014). Application of constructed wetlands for wastewater treatment in developing countries – A review of recent developments (2000–2013). *Journal of Environmental Management*, *141*, 116–131. https://doi.org/10.1016/j.jenvman.2014.03.015

Zhang, Y., Yang, J., Lin, X., Tian, B., Zhang, T. and Ye, S., 2024. Phytoplankton Community Dynamics in Ponds with Diverse Biomanipulation Approaches. *Diversity*, *16*(2), p.75.

Zhou, S., Shao, Y., Gao, N., Deng, Y., Qiao, J., Ou, H., & Deng, J. (2013). Effects of different algaecides on the photosynthetic capacity, cell integrity and microcystin-LR release of *Microcystis aeruginosa*. *Science of The Total Environment*, *463–464*, 111–119. https://doi.org/10.1016/j.scitotenv.2013.05.064

Zou, H., Pan, G., Chen, H., & Yuan, X. (2006). Removal of cyanobacterial blooms in Taihu Lake using local soils II. Effective removal of Microcystis aeruginosa using local soils and sediments modified by chitosan. *Environmental Pollution*, *141*(2), 201–205. https://doi.org/10.1016/j.envpol.2005.08.042
