# Supplementary material for: A Practitioner-Informed Decision Tree for Selecting Harmful Cyanobacteria Bloom Control and Mitigation Techniques
Source: WIREs Water. Author manuscript; Available in PMC 2025 Mar 7. (PMC11887456; doi:10.1002/wat2.70005)
Supplement: Suppl 2 [file NIHMS2060286-supplement-Suppl_2.pdf]

S2. Survey questions

Q1. This survey is being conducted to understand what drives selection of practices for suppressing Harmful Cyanobacterial Blooms, and how those practices vary geographically in the United States. It was developed by Desiree Tullos (OSU), Meghan Skinner (USFWS), Hans Paerl (UNC), and Ellen Preece (CADWR). No identifying information is being collected, unless provided by you, and results will only be summarized as statistical and geographic distributions. Please contact desiree.tullos@oregonstate.edu with questions. Your participation is completely voluntary, and you can withdraw at any time. By checking the box below, you are consenting to participate in this study.

Q2. For each of the practices below, please identify if you consider the practice to be experimental or established.

Photosynthesizers (a variety of peroxide-based chemicals and titanium dioxide)

Metal-based algaecides (e.g. copper, aluminun)

Herbicides (endothall, diquat, flumioxazin, carfentrazone ethyl)

Algal scrubbers

P immobilization (alum, bentonite)

Wetlands for external nutrient reduction

Floating wetlands inside the lake/reservoir

Barley straw

Artificial mixing for destratification

Hypolimnetic oxygenation

Light manipulation

Biomass removal (flocculants)

Filtration

Sonication

Dredging

Q3. For each of the practices below, please identify if you consider the practice to be commonly or uncommonly applied.

Photosynthesizers (a variety of peroxide-based chemicals and titanium dioxide)

Metal-based algaecides (e.g. copper, aluminum)

Herbicides (endothall, diquat, flumioxazin, carfentrazone ethyl)

Algal scrubbers

P immobilization (alum, bentonite)

Wetlands for external nutrient reduction

Floating wetlands inside the lake/reservoir

Barley straw

Artificial mixing for destratification

Hypolimnetic oxygenation

Light manipulation

Biomass removal (flocculants)

Filtration

Sonication

Dredging

Q4. What are the top five factors you consider when selection a treatment practice for a harmful cyanobacterial bloom? (please choose up to five)

- ☐ Alkalinity
- ☐ Baseline nutrient load
- ☐ Nutrient of concern
- ☐ Nutrient source (internal or external)
- ☐ pH
- ☐ Turbidity
- ☐ Cyanobacteria cell density
- ☐ Cyanobacteria community composition
- ☐ Cycles of drawdown/draining and rewetting
- ☐ Dissolved organic matter
- ☐ Dissolved oxygen concentration
- ☐ Cost
- ☐ Lake size
- ☐ Latitude/growing season length
- ☐ Established practice
- ☐ Water body purpose
- ☐ Temperature
- ☐ Mixed or stratified
- ☐ Mixing frequency/residence time
- ☐ Mixing depth
- ☐ Solar irradiance

Group

Q5. In your experience, among the listed algaecides, which are most commonly applied?

- Photosynthesizers (a variety of peroxide-based chemicals and titanium dioxide)
- Metal-based algaecides (e.g. copper, aluminum)
- Herbicides (endothall, diquat, flumioxizan, carefentzone ethyl)
- Other (lime, etc.)
- Do not know

Q6. In thinking about how to suppress harmful cyanobacterial blooms, are there other factors and considerations not included here that you find to be important?

Q7. In your work to suppress harmful cyanobacterial blooms, what single thing would be most useful to you but is not typically available?

Q8. In which state do you currently reside?

Q9. How would you characterize your work role as it is associated with harmful cyanobacterial blooms?

- Regulatory
- Practitioner/water quality manager
- Researcher
- Other

Q10. Any additional comments about this survey or managing Harmful Cyanobacterial Blooms?
